# Alignment of Helical Membrane Protein Sequences Using AlignMe

**DOI:** 10.1371/journal.pone.0057731

**Published:** 2013-03-04

**Authors:** Marcus Stamm, René Staritzbichler, Kamil Khafizov, Lucy R. Forrest

**Affiliations:** Computational Structural Biology Group, Max Planck Institute of Biophysics, Frankfurt am Main, Germany; UMR-S665, INSERM, Université Paris Diderot, INTS, France

## Abstract

Few sequence alignment methods have been designed specifically for integral membrane proteins, even though these important proteins have distinct evolutionary and structural properties that might affect their alignments. Existing approaches typically consider membrane-related information either by using membrane-specific substitution matrices or by assigning distinct penalties for gap creation in transmembrane and non-transmembrane regions. Here, we ask whether favoring matching of predicted transmembrane segments within a standard dynamic programming algorithm can improve the accuracy of pairwise membrane protein sequence alignments. We tested various strategies using a specifically designed program called AlignMe. An updated set of homologous membrane protein structures, called HOMEP2, was used as a reference for optimizing the gap penalties. The best of the membrane-protein optimized approaches were then tested on an independent reference set of membrane protein sequence alignments from the BAliBASE collection. When secondary structure (S) matching was combined with evolutionary information (using a position-specific substitution matrix (P)), in an approach we called AlignMePS, the resultant pairwise alignments were typically among the most accurate over a broad range of sequence similarities when compared to available methods. Matching transmembrane predictions (T), in addition to evolutionary information, and secondary-structure predictions, in an approach called AlignMePST, generally reduces the accuracy of the alignments of closely-related proteins in the BAliBASE set relative to AlignMePS, but may be useful in cases of extremely distantly related proteins for which sequence information is less informative. The open source AlignMe code is available at https://sourceforge.net/projects/alignme/, and at http://www.forrestlab.org, along with an online server and the HOMEP2 data set.

## Introduction

Integral membrane proteins constitute 25–30% of the genes in a given genome [1]–[3] and play crucial roles in cell biology by allowing cells to interact with their environment; accordingly they constitute pharmacological targets for around 50% of active drugs on the market [4], [5]. The study of these proteins is therefore of considerable interest. However, historically it has been very difficult to determine their structures experimentally [6], [7]. Hence, only ∼1000 high-resolution membrane protein structures are so far available in the Protein Data Bank [8], of which ∼400 are unique (http://blanco.biomol.uci.edu/mpstruc/). This situation has motivated many researchers to turn to remote-template homology modeling, in which the unknown structure of a target sequence is modeled on a known (template) structure of a distantly-related protein, in order to gain insights into membrane protein function. Such studies rely on methods for detecting relationships between two proteins, and subsequently, for accurately aligning their sequences; both of these procedures become increasingly difficult as the similarity between the proteins decreases, particularly below ∼20% identity. The membrane-protein-specific multiple-sequence alignment method PRALINE™ [9], for example, manages to recapitulate only ∼40% of the columns in alignments in the BAliBASE membrane protein reference set 7 [10], suggesting that further improvements are needed.

Membrane proteins have distinct properties from their water-soluble counterparts, because of their more complex environment. For example, there are only two major classes of structure: α-helical or β-barrel. Also, the membrane-spanning regions of proteins in the α-helical class have a distinctive hydrophobic character. The latter property inspired the early use of hydropathy profiles for locating transmembrane regions in a sequence [11]. In such profiles, the hydrophobicity, defined according to the values in a particular scale, is plotted as a function of the sequence position, typically using window averaging to smooth out the noise [12]. Hydrophobicity was also the inspiration for a membrane-protein sequence alignment strategy in which the hydropathy profiles themselves are aligned, rather than the corresponding (somewhat less conserved) primary sequences [13]. This strategy also allowed accurate detection of structural similarity between apparently unrelated membrane proteins [14]–[16].

Aside from the hydropathy-based approach, not many sequence alignment methodologies have been specifically developed for, and trained and tested on, membrane proteins. This reflects in part the limited data available for testing. Only recently have a sufficient number of structures become available that a dataset of known membrane protein structural homologues could be constructed to provide knowledge-based reference alignments, as, for example, in the HOMEP dataset [17]. Such datasets have been used to assess methods for aligning membrane protein sequences [17] and for aligning sequences to structures [18]. One of the best of the methods tested on HOMEP, called HMAP, creates pair-wise alignments of profiles representing each sequence [19]. These profiles include information from evolution (in the form of substitution matrices and sequence homologues), structural propensities, and structural relationships [19], but do not explicitly describe the transmembrane location. Complex information about a protein can also be represented in the form of a hidden Markov model (HMM); one of the most readily-available methods used to align such HMMs to one another, HHalign [20] has been shown to produce accurate models of water-soluble proteins, at least in the context of the HHpred structure prediction protocol [21], [22]. However, this approach does not consider membrane-specific information.

Two multiple sequence alignment (MSA) methods, T-Coffee [23] and MUSCLE [24], were also found to produce relatively accurate alignments of pairs of sequences from the HOMEP set [17]; these approaches exclusively include information from evolutionarily-related sequences. Other MSA methods have since been developed, including ProbCons [25], MSAProbs [26], which, like T-Coffee, optimize the consistency of the MSA with pairwise alignments; and PSI-Coffee [27], which aligns each sequence by treating it as a profile. However, none of these methods include membrane-specific information during the alignment. TM-Coffee (a version of PSI-Coffee that uses only membrane protein sequences in the profiles) performs better by some measures than, e.g., MSAProbs for the BAliBASE reference set 7, but is significantly slower [27]. KalignP [28], a recent update of the fast and low-memory usage method Kalign2, can handle position-specific gap penalties, e.g., in transmembrane regions, but is less accurate than ProbCons and T-Coffee on BAliBASE set 7 [28].

Other methodologies developed specifically for membrane proteins include the MSA methods STAM [29] and PRALINE™ [9]. In both cases, the membrane environment is described by using a membrane-protein specific substitution matrix (PHAT) in the transmembrane regions, and a non-specific substitution matrix (BLOSUM62) outside the membrane, although in STAM the transmembrane segments are first separated out and aligned independently, whereas in PRALINE™ the sequences are undivided. Using specific substitution rates that depend on secondary structure, membrane position and solvent accessibility in “membrane FUGUE” improved pairwise sequence-to-structure alignments relative to FUGUE, the equivalent approach for water-soluble proteins [18]. A recent development of membrane FUGUE, called MP-T, incorporates homologues into a MSA in order to guide pairwise sequence-to-structure alignments; MP-T compared well with standard methods on a membrane protein dataset [30].

Here, we use our membrane protein sequence alignment program, AlignMe, to ask whether favoring matching of transmembrane regions – predicted either using hydrophobicity profiles or more sophisticated transmembrane predictions – increases the accuracy of pairwise alignments relative to the matching of secondary-structure elements alone, in the context of a profile-profile type alignment. We also test different methods for treating substitution rates, using either general or position-specific substitution matrices (PSSMs). We update the dataset of homologous helical membrane proteins for optimization and evaluation of the different strategies. We then compare the best of the different AlignMe strategies to other available methods using the BAliBASE membrane protein reference set 7 [10].

## Methods

### 1.1 AlignMe Program Overview

AlignMe (for Alignment of Membrane proteins) is a protein sequence alignment tool developed in C++, which was designed to allow multiple different (membrane) protein descriptors to be considered simultaneously when defining the similarity between two aligned positions. Thus, structural properties such as transmembrane location or secondary structure represented in the form of profiles, can be combined with sequence and evolutionary information represented in the form of substitution rates (see below for more details). The description of the transmembrane location can be a window-averaged hydropathy plot (as used previously [16]), or output from a transmembrane helix predictor.

The underlying algorithm in AlignMe is a Needleman-Wunsch dynamic programming algorithm with affine gap penalties; it is similar to that of BCL::Align [31], although AlignMe allows for more flexible handling of profiles.

The similarity *Sim* between two residues (*i, j*) at a given alignment position is calculated as a linear combination of values from *M* input substitution matrices (*S*), and differences between residue property values (*V*) from *N* input profiles:
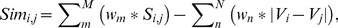
(1)such that any number of substitution matrices can be combined with any number of profiles. Using weights (*w*) for each input is intended to minimize bias towards a specific input. For example, a hydrophobicity scale containing values from –3.0 to 1.0 (i.e., a range of 4.0) would be assigned *w* = 5 when used in combination with a substitution matrix whose values range from –5 to 15 (i.e., a range of 20).

Within AlignMe, gap-opening and gap-extension penalties can be assigned different values according to whether the gap is at a terminus (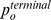 and 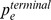, respectively), or not (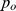 and 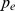, respectively). This flexibility can be useful when aligning sequences whose lengths differ due to additional terminal domains [31]–[33]. In addition, the opening or extension of non-terminal gaps may be assigned different penalties according to an external criterion, such as localization within a hydrophobic or otherwise conserved region, which may be useful for alignments with long internal insertions, because it allows smaller penalties for gaps in those regions. Specifically, given a threshold value for one of the input parameters, the alignment is divided into two regions, i.e., either above or below the threshold. In the case of a hydrophobicity scale, for example, positions with values above the threshold (i.e., hydrophobic) may be assigned different gap penalties (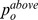 and 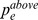) from hydrophilic positions with values below the threshold (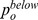 and 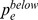). This scheme consequently assigns six gap penalty types in total, namely 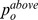, 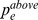, 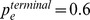, 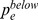, 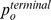 and 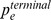.

The source code and manual for AlignMe are provided as Files S3 and S4.

### 1.2 Input Descriptors tested with AlignMe

Differences between inputs were measured using the Wilcoxon signed ranked test [34] and were deemed to be significant when *p*<0.05.

#### Substitution matrices

Several different substitution matrices were compared: BLOSUM62, BLOSUM30 [35], PAM240 [36], VTML [37], [38], JTT membrane version [39], PHAT [40] and bbTM [41]. Unlike BLOSUM, PAM and VTML, these JTT and PHAT matrices were constructed specifically from datasets of α-helical membrane proteins. A BLOSUM-like approach, in which substitutions rates were taken from blocks of transmembrane protein sequence alignments with a certain degree of divergence, was used to generate the JTT matrix [39], whereas the PHAT matrix [40] was constructed from alignments of predicted hydrophobic or transmembrane regions in the BLOCKS+ database. The bbTM matrix was constructed from β-barrel protein sequences [41].

To account for the variability in evolutionary pressure for different positions along the sequence we consider the position-specific substitution rates of the residue types in the two input sequences taken from position-specific substitution matrices (PSSMs). Thus, the similarity is an average of the substitution rate (*S*) at which an amino acid (*A*) from one sequence (*i*) is replaced by the amino acid (*B*) of the other sequence (*j*), and of the rate of the reverse substitution:
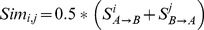
(2)


The PSSMs used as input to AlignMe were those generated during PSIPRED predictions by a PSI-BLAST search on the Uniref90 database dated 28^th^ April 2009.

#### Hydrophobicity scales

Six different hydrophobicity scales were tested. Several were derived from experimental free energies of transfer of amino-acids between ethanol and water [42], including the scales reported by Hopp and Woods (HW) [43] and by Wimley and White (WW) [44]. The Kyte and Doolittle (KD) [11] and the Goldman, Engelman and Steitz (GES) [45] scales were both constructed by combining such transfer free energies with known structural properties or theoretical considerations, while Eisenberg and Weiss (EW) created a consensus of five other scales [46]. White, von Heijne and colleagues (HWvH), derived a hydrophobicity scale from probabilities of α-helical segments inserting into a biological membrane [47], whereas the knowledge-based unified hydrophobicity scale (UHS) [48] was constructed from the distribution of amino acid types in known protein structures.

When using hydrophobicity scales, any position with *V_i_ ≥*0 was assigned to the membrane.

#### Sliding-Window averaging

To generate a smooth hydrophobicity profile, it is typical to replace the value at a given residue with an average over a window of residues centered at that position, and then process that window along the protein sequence [11]. Here, rectangular, triangular or sinusoidal windows of length *L* = 13 were tested [48]. The sinusoidal shape mimics the amphipathic periodicity of a transmembrane helix, so that values 3.6 positions away from the center are given equal weight, while other positions contribute less.

#### Transmembrane helix predictions

Three different predictors for α-helical transmembrane segments were tested: TMHMM [2], OCTOPUS [49] and MEMSAT-SVM [3]. The latter two methods use PSSMs in addition to the raw sequence. These PSSMs were obtained from a PSI-BLAST search against the corresponding recommended database, namely the Uniprot_Sprot database (on 1^st^ August 2010) for MEMSAT-SVM and a version of Uniref90 filtered for transmembrane proteins (from 4^th^ August 2010) for OCTOPUS [49]. The per-residue membrane propensity was used as a profile input for AlignMe. Positions with per-residue propensities >0.5 (for OCTOPUS and TMHMM), or >0 (for MEMSAT-SVM) were defined as being in the membrane.

#### Secondary structure predictions

Two secondary structure predictors were tested: Jufo [50] and PSIPRED [51]. PSI-BLAST searches were run for each method on the corresponding recommended database, i.e., Uniprot_Sprot (from 1^st^ August 2010) and Uniref90 (from 28^th^ April 2009), respectively. Each method produces a three-state prediction of the probability of a position being in a coil, α-helix or β-sheet; all three were used as input profiles for AlignMe, with each state contributing one third of the whole. A position was assigned to an α-helix if the predicted probability thereof was >0.5.

### 1.3 HOMEP2 Training and Test Set

The original HOMEP dataset contained 36 structures [17]; in subsequent years there was a significant increase in the number of available membrane protein structures [52]. To update the database, we introduced a more automated procedure. First, structures and transmembrane definitions were collected from the PDB_TM database (dated 17^th^ March 2010) [53], [54], and filtered to remove NMR structures, theoretical models and structures with resolution >3.5 Å. Individual membrane-spanning chains were extracted and assigned to either α or β subsets, according to PDB_TM. Next, all chains within a subset (α or β) were aligned with all other chains using a structural alignment program SKA [55], [56], unless the two chains belonged to the same PDB entry. For pairs of chains with >85% identical residues (according to the structure-based alignment), only the structure with higher resolution, or smaller R-factor, was retained.

This non-redundant set was then clustered to identify families of related structures. The clustering method (File S1, Figure S1) is based on the protein structure distance (PSD) value that is calculated during SKA structural alignments [55]; here we assume that two proteins are homologous if the PSD <1.2, which is roughly equivalent to belonging to the same superfamily according to the SCOP structural classification scheme [57]. The resultant HOMEP2 data set (File S2) includes 125 structures belonging to 31 structurally distinct families. The subset of α-helical proteins used here contains 81 structures clustered into 22 families containing 177 pair-wise alignments (see File S1, Tables S1 and S2). During cross-validation, 2 of those 22 families were left out in each of 11 repetitions. The structure-based alignments obtained using the SKA program [55] were used as references against which alignment quality on the HOMEP2 set was evaluated (see legend in File S1, Table S2).

### 1.4 Alignment A3ccuracy Measure

The accuracy of a sequence alignment is often evaluated using a score that counts the fraction of correctly aligned positions with respect to the reference alignment [58]. However, this score becomes less useful for more distantly related protein sequences, because it does not discriminate between different degrees of mismatch. Other scores also consider the shift size, defined as the number of positions that a residue in the test alignment is displaced from its aligned column in the reference alignment. For example, the fraction of positions aligned within a certain shift size has been used [19], with the disadvantage that it introduces an arbitrary cut-off in the accuracy measure. In a more advanced strategy, the Cline score penalizes shifts asymptotically, so that it emphasizes residues that are close to their correct position and undervalues errors of greater than four positions [59].

The Alignment Difference (AD) score used here is similar to the mean shift error (MSE) score [60] or the position shift error (PSE) score [61], and takes into account the full extent of any shifts. Residues aligned to other amino acids (and not to gaps) are assigned a score of zero if correctly aligned in the test alignment, whereas shifted positions are penalized by the shift value, as in the MSE score. However, in the AD score, gap-containing columns in the test alignment are treated differently: the shift value of such columns is defined as the mean of the shift values for the two residues either side of the aligned gap. The final AD score is the sum of the (negative) shift values of all columns of the reference alignment divided by its length. Thus, a perfect alignment has an AD score of zero, while more negative values represent less accurate alignments. The AD score correlates with the fraction of correctly aligned positions, but the two measures deviate at low values, and thus the AD score provides distinct information in that realm (File S1, Figure S2a).

### 1.5 Gap Penalty Optimization

A Monte Carlo scheme was used to optimize the gap penalty values for each combination of inputs tested. Note that a systematic optimization [58] is not computationally feasible for optimizing six different gap penalties: if each gap penalty were allowed to range from 0 to 10 in increments of 0.1, a systematic search would require 100^6^ = 10^12^ alignments.

In each step of the optimization process, AlignMe alignments were created using the current set of gap penalties and evaluated using the AD score. To minimize bias towards large families, all proteins in each HOMEP2 family were aligned with all others in that family and their AD scores were averaged; the overall alignment accuracy score for a given set of gap penalty parameters was the sum over the scores for each family.

Starting with a randomly selected set of values (between 0 and 30) for each gap penalty parameter, the search procedure then involved random modifications of one or more gap penalty values from those values, or from the optimal values identified so far. The range of allowed modifications was initially set to be very small (with a maximal step size of 0.06) to encourage a detailed examination of the score landscape around the current optimal gap penalty combination. A given combination of gap penalties was accepted if the overall alignment accuracy score was better than the best score found so far, in which case the maximal step size was reset to its initial value. Otherwise, that combination of gap penalties was rejected and the search space was expanded by increasing the maximum step size by 0.06. However, the gap penalty values were limited to the range 0 to 30, with a maximum step size of 30. If no improvements were found after reaching the maximum step size, the search was repeated, starting with the initial maximal step size of 0.06.

For each set of input descriptors, this optimization was repeated 20 times with different initial gap penalty values, which was found to be sufficient for reasonable convergence (data not shown). The parameters for which the alignments had the best score were then used for that set of input descriptors. The optimal gap penalties obtained using the JTT membrane substitution matrix were 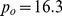, 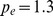, 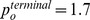 and 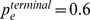, consistent with typical values (e.g. [62]), providing confidence in the optimization procedure.

Optimization of the weights assigned to each input parameter was found to be computationally impractical because the search space increases by the power of *N+M* and the parameters did not converge reliably.

### 1.6 BAliBASE Test Set

Reference 7 set of BAliBASE [10] was used as an independent test set. This set contains 435 membrane proteins in 8 superfamilies, namely 7tm, acr, photo, dtd, ion, msl, Nat and ptga, each multiply aligned. The first three of these families are represented to some extent in the GPCR, multidrug efflux and (bacterio)rhodopsin families, respectively, of HOMEP2 (Table S1). During the evaluation, alignments were generated for all pairs of sequences in the 8 superfamilies. Since we evaluate pairwise sequence alignments, we calculated the fraction of correctly aligned residues as well as the average shift for each alignment, rather than SP (Sum of Pairs) or TC (Total Column) scores, which describe the accuracy of MSAs.

The so-called ‘core’ regions provided by BAliBASE were not analyzed, as they have been shown to correspond only weakly to conserved secondary structure elements in this set [63]. Instead, we analysed segments in each pairwise alignment that were predicted to be transmembrane in both sequences by MEMSAT-SVM; this predictor is the most accurate (see Section 2.1), and using it here avoids bias in the analysis towards one of the alignment methods that uses OCTOPUS (AlignMePST; see Section 2.4).

### 1.7 Alignment Accuracy Tests Based on Homology Models

The accuracy of the alignments in the HOMEP2 set was also assessed by building homology models based on each of the alignments, and comparing them to the native structure. For every pair of protein sequences, each protein was modeled using the structure of the other protein as a template. In each case, five models were created using Modeller v9.9 and the one with the best (lowest) DOPE score was evaluated using GDT_TS (global distance test total score) and AL4 (aligned within four positions) scores [65]. The GDT_TS score is defined as the percentage of Cα atom pairs from the model and the native structure averaged over four different cutoff distances (i.e., 1, 2, 4 and 8 Å) and correlates closely with the percentage of correctly-aligned residues (File S1, Figure S2b). By contrast AL4 considers all positions that are up to 10 Å apart, corresponding to an approximate shift of four alignment positions (File S1, Figure S2c), which allows a clearer discrimination between low-accuracy models than GDT_TS since it is not dominated by the information at the other cutoff levels. For a helical membrane protein, shifts of four positions can still be readily overcome by manual adjustments to the alignment, and thus AL4 describes all residues in a model that may be refined manually.

### 1.8 Other Alignment Methods Tested

Alignments were also calculated with HMAP [19], T-Coffee v8.9.1 [23], MUSCLE v3.7 [24], ProbCons v1.12 [25], MSAProbs v0.9.4 [26] and HHalign v1.5.0 [20]. For MSAs, sequence homologues for each of the sequence were identified using a PSI-BLAST search on the non-redundant (nr) database dated 4^th^ August 2010, with five iterations, an E-value cut-off of 10^−4^ and a maximum of 2500 sequences. Sequences in the PSI-BLAST results that were more than twice the length of the query were filtered out. The remaining sequences were clustered using UCLUST [66] with the original sequence taken as the representative of the first cluster. For T-Coffee, ProbCons and MSAProbs, which are extremely memory- and cpu-intensive, it was necessary to reduce the number of input sequences significantly in order to make the test over the whole HOMEP2 dataset computationally tractable, and so, for all the tested MSA methods (including MUSCLE) we used the suggested T-Coffee protocol, namely selecting the 25 “most–informative” homologues of each sequence (including the query) from the UCLUST clustered results [24].

There are two different possible approaches for generating a MSA from two query sequences and their respective homologues. In the standard approach, all results of both PSI-BLAST searches (including the two query sequences) are combined and aligned as a single large MSA, before extracting out the two query sequences for scoring. The second approach, which we call the “profile-profile” strategy, is to create MSAs for each query and its homologues. The resulting two MSAs or “profiles” are then aligned to one another to create a single MSA, from which the query sequences are then extracted for scoring.

HHalign uses a similar strategy to the MSA “profile-to-profile” approach, but each query is described by a hidden Markov model (HMM) based on the results from a PSI-BLAST search (as for AlignMe PSSMs, see Section 1.2), as well as by secondary structure predictions from PSIPRED, generated as described above. Those HMMs were then globally aligned to each other by using the “-mact 0.0″ maximum accuracy flag and by assigning all other parameters their default values.

HMAP was also used to calculate profile-to-profile alignments. In this case, one of the sequences was assigned to be the query, and its profile included evolutionary information (obtained as for HHalign and AlignMe PSSMs, see Section 1.2) combined with predicted secondary-structure from PSIPRED v3.2; the other sequence was assigned to be the template, and its profile was similar except that the secondary structure was assigned from the structure, where available. The two profiles were then globally aligned using HMAP.

We also tested TM-Coffee [27], but found the computational cost prohibitive for the large number of pairwise alignments in the BAliBASE set (see Section 2.4). STAM, PRALINE™ and MP-T were not available for local installation, and therefore could also not be tested on our large datasets.

## Results

We first describe the selection of input descriptors for AlignMe. To make the comparison between descriptors as fair as possible, gap penalties were optimized for each input tested. To this end, we first constructed an updated set of homologous membrane protein structures (HOMEP2; see Section 1.3), and structure-based sequence alignments of these proteins were used as a reference. Input descriptors were considered to be effective if the AlignMe alignments had both more correctly aligned positions and smaller shift errors, measured as less negative AD scores (see Section 1.4). By also considering the shift error, we expect to help identify methods that are effective for very distantly related proteins. The findings are described for single inputs (Section 2.1) and then for combinations of inputs (Section 2.2). Finally, we compare three of the optimized AlignMe strategies with available alignment programs for the HOMEP2 set (Section 2.3) and the BAliBASE reference set 7 (Section 2.4).

### 2.1 Single Inputs

Various substitution matrices, hydrophobicity scales, secondary-structure and transmembrane predictions were tested as individual inputs (see Section 1.2).

#### Alignment using substitution matrices

**Figure 1:**
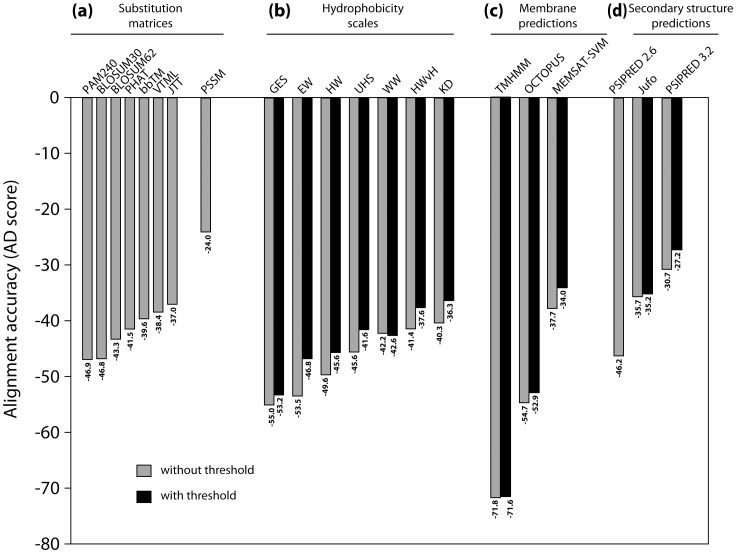
Comparison of alignment accuracy when using single input descriptors in AlignMe. The total alignment accuracy score (AD score) for all α-helical proteins in the HOMEP2 dataset is plotted for each of the input descriptors using their optimized gap penalties, and arranged according to increasing score for different (a) substitution matrices, (b) hydrophobicity scales (with no smoothing), (c) other transmembrane predictions or (d) secondary structure predictions. Sequence segments with hydrophobic, helical or transmembrane scores above a given threshold could be assigned the same (gray bars; without threshold) or different (black bars; with threshold) gap penalty values from segments below that threshold (see Methods for definition of threshold values and abbreviations).

Comparing alignments constructed using different substitution matrices as inputs (Figure 1a) indicates that the alignments in closest agreement with the structure-based reference alignments are obtained using position-specific substitution rates (from PSSMs, see Section 1.2; AD score = –24.0; *p*<10^−9^). Of the general substitution matrices, the closest agreement with the reference alignments was obtained with the membrane-specific JTT matrix, followed by the general-purpose VTML matrix, although the differences between JTT and the others were not very significant (*p* = 0.01 to 0.33; Figure 1a).

#### Alignment based on hydrophobicity scales

Representing sequences by their hydrophobicity values (without averaging their values over a sliding window) is equivalent to using a substitution matrix, except with a focus on one specific physicochemical property. Alignments constructed using hydrophobicity scales were not significantly different from the best of the general substitution matrices (Figure 1b) if all non-terminal gaps were penalized equally (gray bars). The accuracy increased if non-terminal gap penalties were allowed to differ within the transmembrane segments, but again the differences were not statistically significant (Figure 1b, black bars; see Section 1.1). The alignments generated using the KD, HWvH and WW hydrophobicity scales were significantly more accurate (*p*>0.05) than those from other approaches (Figure 1b, black bars), but not significantly different from one another (*p*<0.05). Window-averaging the hydrophobicity values as in a hydropathy plot (File S1, Figure S3) did not significantly improve the alignments compared to using PSSMs (*cf.* Figure 1a).

#### Alignment using transmembrane predictions

More sophisticated predictors of the location of transmembrane helices were also tested. Alignments generated using MEMSAT-SVM alone were not significantly more similar to the reference alignments than those obtained using a hydrophobicity scale or a substitution matrix. The MEMSAT-SVM and OCTOPUS-based alignments became significantly more accurate when penalties were assigned differently to gaps in membrane and non-membrane segments (black bars, Figure 1c), and these MEMSAT-SVM alignments were also significantly (p<10^−4^) more accurate than those generated using the best of the hydrophobicity scales (KD, Figure 1b). Interestingly, the similarity to the reference alignments correlates with the accuracy of the corresponding transmembrane prediction method: MEMSAT-SVM is a significantly more accurate predictor (88.2% of the residues in HOMEP2 are correctly predicted, using PDB_TM assignments as a reference), followed by OCTOPUS (86.4%) and TMHMM (83.0%).

#### Alignment using secondary structure predictions

When representing the sequences as profiles of predicted secondary structure type, the alignments in closest agreement with the reference alignments were obtained using PSIPRED3.2 predictions (Figure 1d). However, only the difference between Jufo and PSIPRED3.2 is statistically significant (*p* = 0.01, gray bars). We note that the other differences are not significant (*p*>0.05) because of a disproportionate contribution of good PSIPRED2.6 alignments in the (large) aquaporin family; this contribution is not reflected in the AD scores in Figure 1 because AD scores are averaged over families (see Section 1.5). Here, again, the alignment accuracy correlates with that of the underlying prediction, with PSIPRED3.2 more accurate (75.3% of residues are correctly predicted) than the other methods tested (74.0% for PSIPRED2.6, and 70.4% for Jufo; *p*<0.05) for the HOMEP2 protein set, using DSSP assignments as a reference [67]. Notably, allowing the penalties for gaps in α-helical structure elements to differ from those in other regions improved the alignments significantly (black bars, Figure 1d).

Comparing all the alignments generated with a single input descriptor, we find that significantly more accurate alignments were obtained using position-specific matrices (PSSMs), followed by secondary structure predictions (PSIPRED3.2, *p* = 2×10^−5^), and transmembrane predictions (MEMSAT-SVM, *p* = 5×10^−6^) (Figure 1). This finding reflects the more detailed information included in the evolutionary profiles compared to the secondary structure and transmembrane predictions.

### 2.2. Alignment using Multiple Input Descriptors

Using the results for single inputs, we next tested alignments for which the best two or three input descriptors were used in combination, since inclusion of complementary information is expected to progressively improve alignment accuracy (see, e.g. [17], [68]).

#### PSSMs combined with a transmembrane prediction

**Figure 2:**
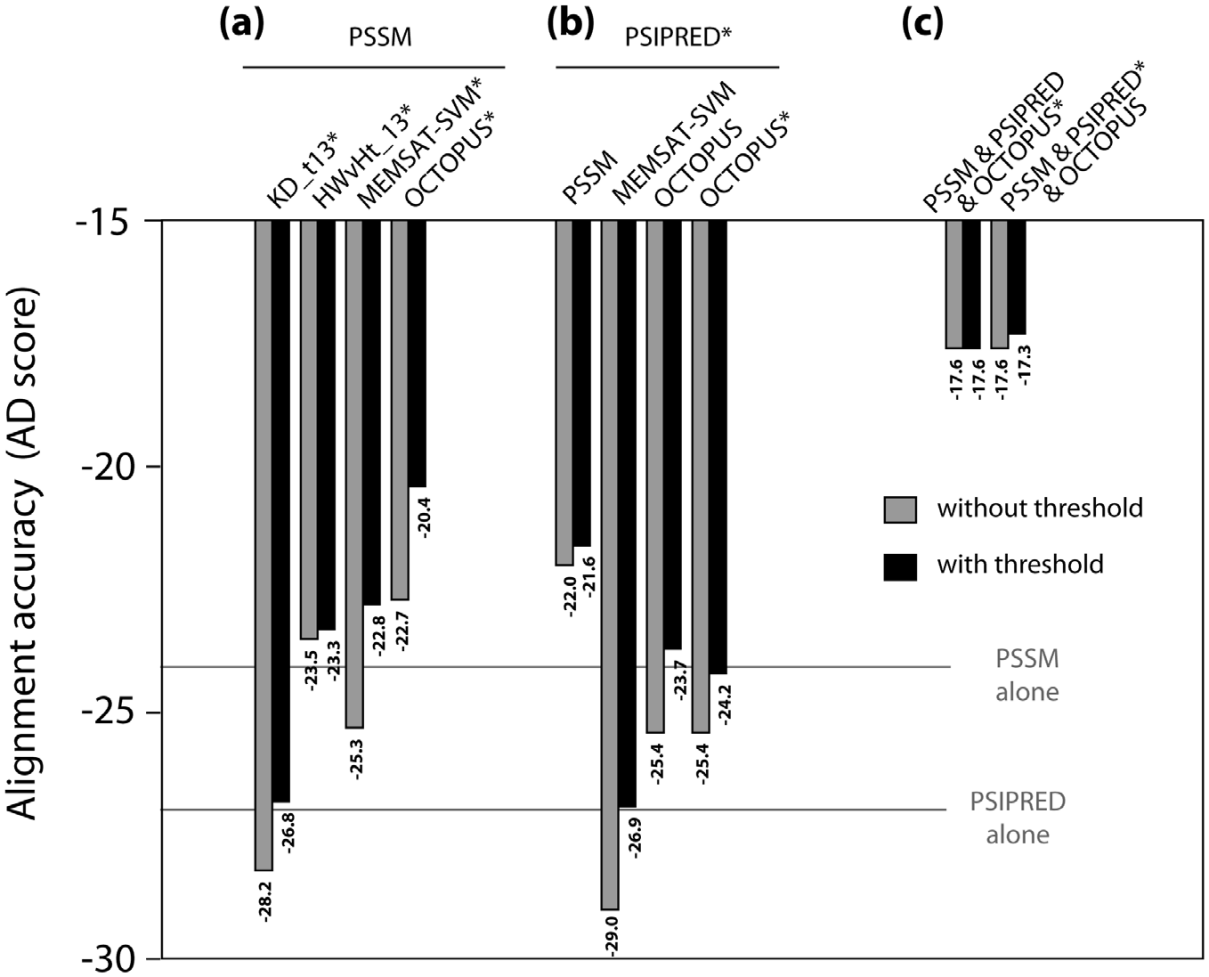
Comparison of alignment accuracy when using multiple input descriptors in AlignMe. Combinations included: (a) PSSMs with hydrophobicity descriptors or transmembrane predictions; (b) secondary structure prediction with PSSMs or transmembrane predictions; or (c) PSSMs, PSIPRED and OCTOPUS together. The scores obtained using PSSMs or PSIPRED alone are indicated with gray lines for reference. Gap penalties were assigned differently to sequence segments above or below a threshold (black bars), and the threshold was defined using the inputs marked by *. For example, in the PSIPRED* & OCTOPUS combination, the threshold was assigned using PSIPRED. See legend for Figure 1 for further details.

A potentially useful combination for membrane proteins is evolutionary information plus transmembrane propensity. The latter can be in the form of either a smoothed hydrophobicity value or a transmembrane prediction propensity. Interestingly, in AlignMe, nearly all such combinations resulted in significantly more accurate alignments than those based on the corresponding individual input parameters, but only when gap penalties were allowed to differ between membrane and non-membrane regions (black bars, Figure 2a). Surprisingly, alignments based on PSSMs were significantly more accurate when combined with OCTOPUS (AD score of –20.4) than with MEMSAT-SVM (AD score of –22.8), even though MEMSAT-SVM predictions are more accurate *per se* (Section 2.1). The explanation could be that OCTOPUS predictions of two related proteins match one another better than those of MEMSAT-SVM, or that the OCTOPUS predictions have a simpler form, perhaps providing more orthogonal (complementary) information to the PSSMs than the more detailed profiles obtained from MEMSAT-SVM (File S1, Figure S4). Alternatively, the fact that the MEMSAT-SVM values are more evenly distributed over a wider range of values than the OCTOPUS scores (File S1, Figure S4) and are thus given a smaller weighting (see Section 1.1) could mean that the MEMSAT-SVM scores can have less influence on the alignments.

#### PSSMs combined with a secondary structure prediction

Combining secondary structure with evolutionary information has been shown to improve profile-to-profile alignments for water-soluble proteins (e.g., [19]). Using AlignMe, a similar improvement is observed in the HOMEP2 alignments when combining PSSMs with PSIPRED predictions: compared to the best results for alignments using secondary structure predictions (AD score of –27.2) or evolutionary information (AD score of –24.0), the combination produces significantly more accurate alignments (AD score of –21.6, *p* = 0.04; Figure 2b, black bars).

#### Secondary structure prediction combined with a transmembrane prediction

Alignments using combinations of a secondary structure prediction with a transmembrane prediction were also significantly more accurate (AD score of –23.7 for PSIPRED combined with OCTOPUS; Figure 2b) than alignments using each descriptor on its own (AD score of –27.2 for PSIPRED and –52.9 for OCTOPUS; Figure 1), with OCTOPUS again being the best choice of transmembrane predictor (Figure 2b). This observation suggests that secondary structure and transmembrane predictions contain complementary information, consistent with the fact that not all secondary structure elements in a membrane protein are within the membrane. Indeed, among the 60% of the residues that are outside the membrane in HOMEP2 structures (as defined by PDB_TM), 46.2% of residues are α-helical, and 7.4% are in a β-strand. Moreover, not all transmembrane segments are fully helical [69], and include segments of coil (7.5% of residues) and even β-sheet (0.1% of residues).

In these combinations, when assigning gap penalties differently to structured regions (Section 1.1), the latter may be defined using either secondary structure or membrane propensity. We found that using α-helix positions for this distinction (OCTOPUS and PSIPRED*, AD score of –23.7, Figure 2b) led to significantly (*p* = 0.03) more accurate alignments than when using the transmembrane positions for assigning the thresholds (OCTOPUS* and PSIPRED, AD score of –24.2, Figure 2b). This makes sense because gap insertion should be disfavored in all structured regions, whether in the membrane or not. Nevertheless, no matter how the different regions are assigned, the alignments are significantly more accurate than when the same gap penalty values are used in both structured and unstructured regions (Figure 2b).

#### Combinations of PSSMs with secondary structure and transmembrane predictions

The three protein descriptors with the most useful and complementary information (PSSM, OCTOPUS and PSIPRED3.2) were next tested in combination, which resulted in significantly more accurate alignments than any approach tested so far (AD score = –17.6, *p*<0.05, Figure 2c). A further significant increase was obtained by assigning gap penalties according to secondary structure propensity (AD score = –17.3, *p*<0.05, Figure 2c), but not by transmembrane position (AD score = –17.6, *p* = 0.32, Figure 2c).

Interestingly, the input descriptors that led to the most accurate alignments when used alone were not always the most effective when used in combination (e.g., OCTOPUS contributed more in combination than alone, and the converse was found for MEMSAT-SVM; Figures 1 and 2), presumably because a single input may need to contain detailed information to produce an accurate alignment, whereas in combination that information may become redundant or even conflicting. Clearly this suggests that it would be desirable to optimize the parameters on all combinations of all descriptors, but unfortunately this is not computationally tractable at this time.

In subsequent evaluations we compared three different versions of AlignMe, in each case using gap penalties optimized for the respective combination on the HOMEP2 membrane protein dataset. For reference, we tested one version, called AlignMeP, that uses only evolutionary information (PSSM), with gap penalties of 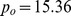, 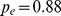, 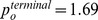 and 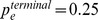. In the AlignMePS version, secondary structure information (PSIPRED3.2) was also used, with gap penalties of 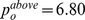, 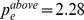, 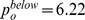, 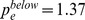, 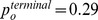 and 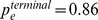. Finally, we tested the effect of including transmembrane information within AlignMePST, where PSSMs, PSIPRED3.2 and OCTOPUS are combined, and using gap penalties of 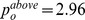, 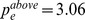, 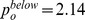, 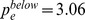, 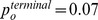 and 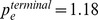. In both AlignMePS and AlignMePST versions, α-helicity was used to define the gap penalty assignment threshold.

### 2.3 Comparison with Other Alignment Methods on the HOMEP2 Dataset

We compared the three AlignMe versions, i.e., with and without secondary structure or membrane matching, to several available multiple-sequence alignment programs, as well as the profile-to-profile alignment program HMAP, and the HMM alignment program HHalign (see Section 1.8). Here, we assess alignments of the training HOMEP2 dataset, first using the structure-based alignments as a reference, and then using the accuracy of homology models built from those alignments as a reference-independent measure.

#### Alignment accuracy relative to structure-based reference alignments

For close homologues (>30% identical residues) in the HOMEP2 set, the AlignMe alignments are not significantly more accurate than other methods: AlignMeP, AlignMePS, AlignMePST and HHalign alignments all have a high fraction of correctly aligned residues, for example (Table 1 and File S1, Figure S5e), and the average shift error is similarly low for HHalign, MSAProbs, AlignMeP and AlignMePST alignments (Table 1 and File S1, Figure S5f). However, for pairs of membrane protein sequences in the HOMEP2 set with low (0–15%) and moderate (15–30%) similarity, AlignMe alignments have ∼2% more correctly-aligned positions than all other methods (Table 1 and File S1, Figures S5a, S5c), which is perhaps unsurprising given that the gap penalties are optimized for this HOMEP2 dataset. The most accurate of the other methods by this measure are MSAProbs and HMAP (Table 1).

**Table 1:** Accuracy of alignments generated using different methods on the HOMEP2 data set.

	0–15% (44)		15–30% (71)		30–85% (62)	
	% correct	shift	% correct	shift	% correct	shift
AlignMeP	30.1*	4.31	**72.0**	1.15	**88.2**	0.25*
AlignMePS	30.6*	3.35	**71.5**	1.16	87.9*	0.28
AlignMePST	**30.7**	**2.73**	70.4	**0.85**	87.5	**0.21**
AlignMePST x-fold	30.3	2.89	70.4	0.89	87.3	0.30
MSAProbs	28.3	7.22	68.6	1.08	85.7	0.24*
HHalign	17.3	10.50	61.8	1.75	86.5*	0.29*
HMAP	24.9	7.00	68.6	1.27	85.3	0.32
MUSCLE	26.4	9.41	68.5	1.13	85.5	0.31
Muscle profile-profile	25.6	9.77	63.6	1.65	75.6	0.86
ProbCons	26.7	8.30	67.0	1.34	84.2	0.31
T-Coffee	25.3	7.55	66.5	1.27	83.4	0.32
T-Coffee profile-profile	14.5	35.22	55.9	2.25	70.7	1.09

Results are sorted according to the level of sequence similarity of the sequence pair, in percentage identity. The number of pairwise alignments is shown in parentheses. The percentage of correctly aligned residues (% correct) and average shift error size (shift) with respect to the structure-based reference alignments (see Methods) are reported. *Values marked with an asterisk in this and all other tables are not significantly different from those of AlignMePST (p-value >0.05) based on a pairwise Wilcoxon signed rank test. All other values are significantly different from those of AlignMePST. Entries in bold in this table, and all subsequent tables, indicate the highest or best scores in that column, including all values that are not significantly different from the best scores.

Misaligned residues are shifted by significantly fewer positions in AlignMe alignments (Table 1 and File S1, Figures S5b, S5d), particularly when transmembrane information is included (see AlignMePST in Table 1), reflecting the optimization of the gap penalties to the shift-size sensitive AD score. We note that the reduction in shift error obtained by matching transmembrane predictions (AlignMePST *cf.* AlignMePS) does come at the cost of some correctly-aligned positions, especially for sequences with moderate similarity. As mentioned above, for homology modeling of distantly-related pairs of proteins it can be useful to reduce the magnitude of large shift errors since manual adjustment of an alignment can be aided relatively easily by conservation mapping once the helices are approximately aligned. For similar reasons, it is also interesting to know whether the transmembrane helices have been matched to some extent, as many (although not all) functional residues lie in these regions. The matching of transmembrane helices in the HOMEP2 set by AlignMe appears to be particularly effective: using AlignMePS and AlignMePST, ≥97% of the known transmembrane helices overlap by at least half of their residues, and ≥62% of the helices (at least 10% more than the next best method) overlap by at least 90% of their residues (Table 2). These enhancements are achieved largely because of the inclusion of secondary structure information (compare AlignMePS to AlignMeP), and to some extent by the matching of transmembrane predictions (compare AlignMePS to AlignMePST). However, even without transmembrane predictions, AlignMePS also matches these segments significantly better (8–12% more segments of distant homologs (0–15%) overlap by at least half of their residues) than another method that considers secondary structure (HMAP), at least on the HOMEP2 training set.

**Table 2:** Percentage of transmembrane segments in the HOMEP2 set that are correctly aligned by each method.

	0–15% (44)		15–30% (71)		30–85% (62)	
	*f* ^50^	*f* ^90^	*f* ^50^	*f* ^90^	*f* ^50^	*f* ^90^
AlignMeP	93.65	52.80	98.64	95.54	100.00*	99.31*
AlignMePS	97.00	62.43*	99.49*	96.85*	100.00*	99.08*
AlignMePST	**98.32**	**63.73**	**100.00**	**97.17**	**100.00**	**99.77**
MSAProbs	90.42	53.01	99.49*	95.90*	100.00*	99.31*
HHalign	70.50	28.61	97.05	76.97	100.00*	95.72*
HMAP	85.83	54.31	99.49*	96.87*	100.00*	99.08*
MUSCLE	82.92	49.59	99.60*	93.89	100.00*	99.04
MUSCLE profile-profile	82.20	48.90	98.08	86.30	99.46*	88.16
ProbCons	89.73	52.17	99.49*	95.78*	100.00*	99.04
T-Coffee	88.02	51.18	99.49*	95.42	100.00*	98.85
T-Coffee profile-profile	38.32	18.75	95.56	66.68	97.33	73.12

Transmembrane segment definitions are taken from the structures according to the PDB_TM database (see Methods); matching is defined as correct if 50% (*f* ^50^) or 90% (*f* ^90^) of the residues are aligned. Results are sorted according to the level of sequence similarity of the sequence pair. The number of pairwise alignments is shown in parentheses.

#### Cross-validation

An obvious concern regarding the robustness of the AlignMe method(s) is overtraining of the gap penalties and other parameters due to the limited number of membrane protein structures available. We first tested this by using cross-validation: the optimization of AlignMePST was repeated using 20 of the 22 families and the resultant gap penalties used to evaluate the alignment accuracy of the remaining two families. As shown in Table 1 (see x-fold), the accuracy of the alignments using these gap penalties was similar to that obtained by training and testing on the whole HOMEP2 set. Moreover, the mean and standard deviation of the gap penalties for the AlignMePST combination indicates relatively small variations between cross-folds (i.e., after optimization on different subsets): 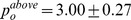, 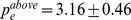, 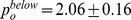, 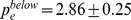, 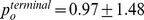 and 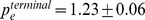. These results suggest that the gap penalties are not significantly over-trained on a particular family of the HOMEP2 dataset, and thus should be applicable to other membrane protein sequences.

#### Model quality

As a measure of alignment accuracy that is independent of the reference alignments, we also constructed structural (homology) models from the test alignments. The models were compared to the native structures by calculating GDT_TS and AL4 values (see Section 1.7), and also compared to “gold standard” models built using sequence alignments extracted from the SKA structural alignments.

Measured by the GDT_TS structural similarity score, several methods have similar accuracy on average (Table 3 and File S1, Figure S6). AlignMe alignments result in fewer very poor models (with GDT_TS <20%), while all other methods produce models with GDT_TS as low as 5% for distantly related proteins of the HOMEP2 set (File S1, Figure S6a). Using the AL4 score, which discriminates better between low-accuracy models (see File S1, Figure S6b), models based on AlignMePS and AlignMePST alignments have up to 5% higher scores than the best of the other alignment methods (Table 3). This result reflects the low average shift error of the underlying alignments (*cf.* Table 1) due to the optimization towards less negative AD scores. We note that models built from the structure-based alignments are the most accurate (SKA; Table 3 and File S1, Figure S6), indicating that there remains room for improvement in alignment methods.

**Table 3:** Accuracy of homology models constructed based on HOMEP2 data set alignments from different methods.

	0–15% (88)		15–30% (142)		30–85% (124)	
	GDT_TS	AL4	GDT_TS	AL4	GDT_TS	AL4
AlignMeP	34.74	73.97	67.53*	90.75	83.94*	97.65
AlignMePS	**38.06**	79.97*	67.40*	90.52	83.79*	97.33
AlignMePST	36.30	**80.48**	**67.36**	**92.19**	**83.96**	**98.03**
MSAProbs	36.71*	75.00	67.33*	90.81	84.17*	97.76
HHalign	25.08	59.06	61.38	87.71	83.12	97.63
HMAP	36.33*	74.97	67.31*	90.44	83.25	97.04
MUSCLE	32.95	69.02	66.00	90.66	82.89	97.31
Muscle profile-profile	32.56	69.35	62.19	88.82	75.75	94.24
ProbCons	35.28*	72.78	67.16*	90.22	83.29	97.46
T-Coffee	35.30*	72.20	66.78	90.42	83.38	97.57
T-Coffee profile-profile	18.27	37.85	59.30	86.58	73.03	92.95
SKA structure-based^a^	46.38	85.42	71.12	93.99	85.51	98.18*

a Reference alignments generated by the structure alignment program, SKA. The number of models is shown in parentheses.

### 2.4 Comparison with Other Alignment Methods using the BAliBASE Reference 7 Set

The alignment accuracy of the various methods was also evaluated on an independent data set of membrane protein sequences (reference set 7 of BAliBASE; see Section 1.6). This dataset contains manually-curated multiple-sequence alignments, based on PFAM alignments and optimized to improve amino acid and secondary structure matching; no structural information was available at the time to help guide the dataset construction [10]. Here, we analyze the accuracy of all pairwise alignments in BAliBASE, and then separate out the results for closely and distantly-related proteins.

AlignMePS alignments have the most correctly aligned residues in BAliBASE set 7 on average, as well as for 7 of the 8 families (Table 4), including those not represented in the training set. Favoring matching of transmembrane segments with AlignMePST results in significantly more accurate alignments for the ion family, which has a very low average sequence identity (Table 4). The AlignMePST alignments also rank second overall, and for the 7tm, dtd and ptga families. Among the other methods, HMAP alignments are also very accurate for this dataset, with the third highest-ranking scores on average, and high-ranking scores for four out of the eight families.

**Table 4:** Percentage of residues that are correctly aligned in pairwise sequence alignments from the BAliBASE reference set 7, sorted by sequence identity of the protein families.

	ion	Nat	ptga	7tm	dtd	acr	photo	msl	mean
AlignMeP	38.9	43.5	42.1	42.5	67.1	87.0	**87.9**	**82.5**	61.4
AlignMePS	45.2	**66.2**	**64.8**	**65.9**	**76.0**	**89.7**	87.6	**82.3**	**72.2**
AlignMePST	**48.1**	58.6	58.8	59.4	71.2	86.3	82.9	76.5	67.7
MSAProbs	24.5	53.3	45.9	54.7	64.4	89.0	73.4	70.6	59.5
HHalign	39.1	48.9	42.3	38.4	42.7	49.5	67.3	59.9	48.5
HMAP	32.8	61.9	54.9	61.4	65.3	87.6	83.4	78.5	65.7
MUSCLE	27.9	56.8*	48.4	56.6	70.3	89.5	80.5	76.1*	46.7
MUSCLE profile-profile	18.5	47.1	39.7	48.2	67.4	88.5	70.4	64.1	55.5
ProbCons	23.8	52.0	44.1	54.4	63.7	88.7	69.3	66.8	57.9
T-Coffee	25.5	50.6	44.2	55.1	63.7	88.8	67.5	67.5	57.9
T-Coffee profile-profile	10.8	14.5	27.0	40.2	52.9	86.2	52.1	53.0	42.1
Number^a^	1326	1711	1275	8128	1485	903	528	91	
Sequence identity (%)^b^	11.7±13.8	14.3±10.8	15.9±12.1	18.2±9.7	18.7±11.5	26.9±11.3	27.3±16.9	35.3±13.5	

Mean = mean percentage of correctly-aligned residues over averages for eight families. ^a^Number of pair-wise alignments. ^b^Mean (±standard deviation) of the percentage sequence identity between pairs of alignments in each family.

To assess whether the inclusion of transmembrane information is useful for distantly-related proteins, we separated the BAliBASE set into sequences assigned to the same subgroup (Table 5) or to different subgroups (Table 6) [10]. The high ranking of the various AlignMe methods and of HMAP remains for alignments of both closely and distantly-related sequence pairs (Tables 5 and 6). For pairs of proteins in the same subgroup, the AlignMePS alignments are significantly more accurate on average (Table 5), but matching of secondary structure is not always beneficial in those cases: indeed, AlignMeP alignments are significantly more accurate for the most similar sequences (in the dtd and photo families, Table 5). Nevertheless, secondary structure and transmembrane information becomes progressively more useful as the similarity decreases, especially for those assigned to different subgroups (Table 6).

**Table 5:** Percentage of residues that are correctly aligned in pairwise sequence alignments assigned to the same subgroup within the BAliBASE reference set 7, sorted by sequence identity of the alignments in each protein family.

	ion	ptga	7tm	Nat	acr	msl	dtd	photo	mean
AlignMeP	62.8	83.4	67.6	80.6	93.4	**82.0**	**90.3**	**94.7**	81.8
AlignMePS	**64.9**	**83.9**	**74.2**	**81.8**	**93.9**	**81.7**	89.6	94.0	**83.0**
AlignMePST	62.9	81.7	68.4	79.3	92.4	78.3	86.9	91.4	80.2
MSAProbs	44.3	67.5	62.5	71.1	92.5*	74.4	84.5	88.8	73.2
HHalign	51.6	52.0	43.9	64.8	56.0	58.6	66.4	84.3	59.7
HMAP	50.6	75.2	69.2*	77.5*	91.7	**80.9**	82.8	90.6*	77.3
MUSCLE	47.0	72.3	62.6	72.4	93.0	78.0*	85.0	88.6	74.9
MUSCLE profile-profile	25.1	60.8	53.5	54.3	91.6	62.6	74.7	74.1	62.1
ProbCons	43.8	66.5	62.1	69.7	92.2	69.9	83.7	83.6	71.4
T-Coffee	45.9	69.8	64.7	72.5	92.2	76.8	85.2	87.0	74.3
T-Coffee profile-profile	45.3	66.3	63.5	70.4	92.1	71.4	84.1	83.6	72.1
Number	551	559	1082	282	420	51	84	122	
Sequence identity (%)	22.1±16.6	26.7±11.0	28.0±20.0	31.3±16.7	34.4±12.9	43.6±12.7	49.5±19.1	52.2±18.1	

See legend to Table 4 for more details.

**Table 6:** Percentage of residues that are correctly aligned in pairwise sequence alignments assigned to different subgroups within the BAliBASE reference set 7, sorted by sequence identity of the alignments in each protein family.

	ion	ptga	Nat	7tm	dtd	photo	acr	msl	mean
AlignMeP	21.9	9.9	36.2	38.6	65.7	**85.9**	81.4	**83.3**	52.9
AlignMePS	31.2	**49.9**	**63.1**	**64.7**	**75.2**	**85.7**	86.0	**83.0**	**67.3**
AlignMePST	**37.5**	41.0	54.5	58.0	70.3	80.3	81.0	74.2	62.1
MSAProbs	10.5	29.0	49.8	53.5	63.2	68.8	85.9	65.9	53.3
HHalign	30.2	34.8	45.8	37.6	41.3	62.2	43.8	61.6	44.6
HMAP	20.1	39.2	58.9	60.2	64.3	81.3	83.9	75.5*	60.4
MUSCLE	14.3	29.8	53.7	55.7	69.4*	78.1	**86.5**	73.7*	57.7
MUSCLE profile-profile	13.7	23.2	45.7	47.4	67.0	69.3	85.7	66.1	52.3
ProbCons	9.5	26.6	48.6	53.2	62.5	65.0	85.8	62.9	51.7
T-Coffee	13.5	34.3	46.5	55.3	63.6	72.8	86.1	69.7	55.2
T-Coffee profile-profile	11.5	26.9	46.7	53.8	62.5	62.6	85.9	62.7	51.6
Number	775	716	1429	7046	1401	406	483	40	
Sequence identity (%)	4.3±1.0	7.5±1.6	10.9±3.9	16.7±5.4	16.8±7.6	19.8±5.5	20.4±1.6	24.7±3.3	

See legend to Table 4 for more details.

Within the predicted transmembrane regions of the BAliBASE sequences (Table 7), AlignMePS alignments have the highest proportion of correctly-aligned positions for four out of the eight families, and the secondary-structure prediction significantly improves the accuracy relative to the AlignMeP alignments. Surprisingly though, the transmembrane information included in AlignMePST does not help to correctly align more positions in the predicted transmembrane regions of the alignments, even for the most distantly-related family (ion; Table 7), despite the fact that AlignMePST aligns more positions correctly over the full length of those sequences (ion; Table 4). This result suggests that secondary structure elements not in the membrane are correctly guided to the appropriate places in the alignment by the transmembrane predictions, but that within the transmembrane regions, the secondary structure and transmembrane predictions conflict with one another, resulting in slight errors in the TM segments (consistent with the small shift errors in these alignments; see below, Tables 8,9,10). The methods that correctly align the most positions in the predicted transmembrane regions are MSAProbs, Probcons, HMAP and T-coffee, depending on the sequence family (Table 7); MSAProbs and AlignMePS alignments are the highest ranking on average (Table 7).

**Table 7:** Percentage of residues that are correctly aligned in the predicted transmembrane regions of pairwise sequence alignments from the BAliBASE reference set 7, sorted by protein family name.

	7tm	acr	dtd	ion	msl	Nat	photo	ptga	mean
AlignMeP	54.6	96.0	76.5	36.1	96.7	44.6	91.8	40.3	67.1
AlignMePS	92.6	**98.0**	**90.1**	58.3	**97.1**	**73.6**	**96.0**	67.2	84.1
AlignMePST	87.0	95.6	86.2	57.8	95.7	64.2	93.9	58.1	79.8
MSAProbs	**95.8**	98.0	89.5	62.7	**96.5**	69.5	91.7	**72.3**	**84.5**
HHalign	51.9	37.6	51.8	37.1	76.3	50.0	71.6	31.5	51.0
HMAP	95.1	97.6	82.8	61.5	96.0*	72.4	**96.7**	69.3	83.9
MUSCLE	89.5	97.6	89.1	49.7	95.0*	64.9	91.7	57.2	79.3
MUSCLE profile-profile	79.9	97.4	89.0	30.2	92.9	53.9	85.8	47.6	72.1
ProbCons	95.7	97.9	89.6	61.6	**96.5**	67.9	90.6	69.8	83.7
T-Coffee	95.8	**98.1**	89.9	**65.7**	**96.4**	66.5	88.2	69.8	83.8
T-Coffee profile-profile	75.5	98.0	83.9	12.3	91.2	18.2	71.8	49.0	62.5

Mean = mean over averages for eight families.

**Table 8:** Average shift error in pairwise alignments of the BAliBASE reference set 7.

	ion	Nat	ptga	7tm	dtd	acr	photo	msl	mean
AlignMeP	29.92	48.71	33.98	47.58	9.83	1.09	0.31*	0.59*	15.38
AlignMePS	28.83	2.46	**3.12**	**3.67**	**1.71**	**0.33**	0.36	**0.42**	5.11
AlignMePST	**13.83**	3.24	5.39	11.82	3.46	0.42	**0.31**	0.47	**4.87**
MSAProbs	37.00	2.42*	5.99	5.17	4.29	0.34	1.36	0.84	6.87
HHalign	15.89	4.81	7.96	9.91	6.37	1.61	0.84	1.78	6.15
HMAP	35.66	**1.95**	6.18	4.61	6.84	**0.31**	0.52	0.58	7.08
MUSCLE	49.39	6.01	12.97	10.42	3.31	0.34	0.73	0.64	10.48
MUSCLE profile-profile	57.33	11.53	18.23	22.06	3.86	0.40	1.28	1.20	14.49
ProbCons	41.46	3.20	7.91	5.60	4.78	0.35*	1.70	1.09	8.22
T-Coffee	39.93	4.62	6.69	4.50	4.73	0.35*	1.60	1.09	7.90
T-Coffee profile-profile	64.15	42.50	12.03	17.50	8.48	0.45	2.15	2.22	18.69

Families are sorted by the average sequence identity (see Table 4). Mean = mean over averages for eight families.

**Table 9:** Average shift error in pairwise alignments assigned to the same subgroup within the BAliBASE reference set 7.

	ion	ptga	7tm	Nat	acr	msl	dtd	photo	mean
AlignMeP	12.35	0.79	16.19	1.45*	0.16*	0.72	**0.62**	0.18	4.06
AlignMePS	6.69	0.73	**2.38**	**1.35**	**0.16**	0.46*	0.58*	0.20*	**1.57**
AlignMePST	**5.57**	**0.65**	8.44	1.45	0.16	**0.44**	**1.09**	**0.16**	2.24
MSAProbs	21.91	2.97	3.90	1.85	0.19	0.70	1.25	0.48	4.16
HHalign	6.03	3.14	8.56	2.37	1.32	1.94	2.66	0.29	3.29
HMAP	17.91	2.03	2.93	**1.40**	0.20	0.55*	3.96	0.26	3.66
MUSCLE	17.67	5.73	9.13	3.56	0.19	0.63	0.99	0.37	4.78
MUSCLE profile-profile	42.37	8.06	15.17	10.81	0.23	1.31	2.36	1.01	10.16
ProbCons	23.82	3.98	4.40	2.44	0.22	1.01	1.55	0.65	4.76
T-Coffee	19.90	1.98	3.42	2.23	0.21	0.58	1.08	0.56	3.74
T-Coffee profile-profile	23.86	3.11	3.62	2.66	0.22	0.79	1.13	0.62	4.50

Families are sorted by the average sequence identity (see Table 5). Mean = mean over averages for eight families.

**Table 10:** Average shift error in pairwise alignments assigned to different subgroups within the BAliBASE reference set 7.

	ion	ptga	Nat	7tm	dtd	photo	acr	msl	mean
AlignMeP	42.41	59.90	58.04	52.40	10.38	**0.35**	1.90	**0.43**	28.23
AlignMePS	44.56	**4.99**	**2.67**	**3.87**	**1.78**	0.40	0.48	**0.35**	7.39
AlignMePST	**19.71**	9.10	3.60	12.34	3.61	0.35	0.65	0.50	**6.23**
MSAProbs	47.73	8.35	2.53	5.37	4.47	1.62	0.47	1.01	8.94
HHalign	22.90	11.73	5.29	10.12	6.60	1.01	1.86	1.56	7.63
HMAP	48.28	9.43	2.06	4.87	7.01	0.60	**0.41**	0.60*	9.16
MUSCLE	71.94	18.63	6.49	10.61	3.45	0.83	0.48	0.65	14.13
MUSCLE profile-profile	67.96	26.17	11.67	23.11	3.95	1.36	0.55	1.05	16.98
ProbCons	54.01	10.98	3.35	5.81	4.97	2.01	0.46	1.20	10.35
T-Coffee	34.91	5.23	4.90	4.32	4.05	1.67	0.44	0.75	7.03
T-Coffee profile-profile	51.36	9.48	5.01	4.62	4.95	1.90	0.47	1.46	9.91

Families are sorted by the average sequence identity (see Table 6). Mean = mean over averages for eight families.

Finally, we also calculated the shift error in the BAliBASE alignments, which is smallest on average for the AlignMePST and AlignMePS alignments, followed by MSAProbs and HHalign, while HMAP has the smallest shift error for the acr and Nat families (Table 8). Matching of secondary structure predictions significantly reduces the shift error relative to AlignMeP for proteins in the same subgroup (Table 9) or in different subgroups (Table 10), whereas transmembrane matching in AlignMePST has the biggest positive influence on the most distantly-related sequences (Table 10), particularly on the alignments in the ion family (Tables 8,9,10).

## Discussion

In this work, we developed a sequence alignment method called AlignMe, which we trained on a dataset of membrane protein structural homologues (HOMEP2). Three different strategies (AlignMeP, AlignMePS and AlignMePST) were assessed using the BAliBASE membrane protein dataset (set 7), and compared with other available methods. Overall, the BAliBASE analysis suggests that versions of AlignMe that match secondary structure prediction profiles may be generally useful for aligning membrane proteins (AlignMePS and AlignMePST; Tables 4,5,6,7,8,9,10). AlignMePS alignments are more accurate than those of HMAP and HHalign, both of which also use secondary-structure information directly, indicating that training AlignMePS specifically on a membrane protein dataset was also advantageous. The additional matching of transmembrane prediction profiles, however, does not improve alignments of closely-related BAliBASE sequences, i.e., AlignMePS results in more accurate alignments for those proteins than AlignMePST. We checked that the transmembrane profiles are indeed matched in the AlignMePST alignments by calculating the difference in OCTOPUS profile values at every position in each alignment, normalizing the total difference by the alignment length, and summing over all HOMEP2 alignments. This profile difference measure is smaller (0.056) when using the transmembrane predictions in AlignMePST than without (in AlignMePS; 0.085), confirming that the predicted transmembrane profiles match more closely in AlignMePST alignments. The fact that transmembrane matching does not improve alignment accuracy for the closely-related BAliBASE sequence pairs may reflect the errors of >10% in the transmembrane predictions (see Section 2.1). Indeed, the matching of OCTOPUS predictions in the reference structure-based alignments is almost as poor (profile difference score of 0.079) as the matching in the AlignMePS alignments. Such prediction errors can potentially be cancelled out in the context of a sequence alignment if the predictions for both sequences are incorrect in the same way, but this is not always the case, and the likelihood of errors canceling diminishes as the sequences diverge in similarity.

As mentioned above, another source of errors for the AlignMePST strategy (especially in the transmembrane regions) may be discrepancies between the secondary-structure and transmembrane predictions. Quantifying the matching of secondary-structure prediction profiles as described above indicates that the secondary structure profiles match less well in alignments generated with transmembrane predictions (profile difference score for AlignMePST is 0.060) than those without (profile difference score for AlignMePS is 0.055). In other words, transmembrane matching occurs at the expense of secondary-structure matching.

A third possible cause of the reduced accuracy for closely-related sequences using AlignMePST is that including a third parameter (the transmembrane prediction) in the score for each position diminishes the contribution of the PSSM in a deleterious way.

The above discussion notwithstanding, the BAliBASE results indicate that incorporating transmembrane matching is useful for very distantly-related proteins, particularly for reducing the overall shift error (Tables 1, 3, 6 and 8,9,10). The observations for the accuracy in the transmembrane segments, however, are somewhat contradictory: the overlap of the known transmembrane regions in the HOMEP2 alignments is increased significantly by including transmembrane profiles (Table 2), whereas in the predicted transmembrane regions of the BAliBASE alignments there were fewer correctly aligned positions than with, e.g. T-Coffee (Table 7). Again, this may reflect conflicts between the secondary structure and transmembrane predictions, which might be addressed in future by adjusting the procedure so that secondary structure information is used only in regions not predicted to be in the membrane. Unfortunately, we do not yet have sufficient data at low sequence identities to test this hypothesis more thoroughly and must await the availability of larger reference sets.

Of the other available methods tested, HMAP alignments were most frequently ranked towards the top (Tables 1,2,3,4,5,6,7,8,9,10), and T-Coffee and MSAProbs alignments were frequently very accurate, particularly in the transmembrane regions of the BAliBASE set (Table 7). Recently, MSAProbs and ProbCons were tested on this same BAliBASE reference 7 set [27]; however, in that study, they were assessed for their ability to construct MSAs rather than pair-wise alignments, which are the focus here. It should also be reiterated that when testing the MSA methods on BAliBASE, we did not construct a single MSA containing only the BAliBASE sequences, but rather, for each pair of sequences, we aligned all homologues of those sequences identified by PSI-BLAST, in order to make the results comparable to those of AlignMe, HHalign and HMAP (see Section 1.8). A consequence of this approach was that TM-Coffee, a slower method also shown to perform well for MSA of BAliBASE set 7 [27], was too computationally expensive to test in the current study.

The profile-to-profile alignments strategy used with MUSCLE and T-Coffee typically resulted in fewer correctly-aligned positions and larger shifts than the other methods tested (Tables 1,2,3,4,5,6,7,8,9,10). HHalign alignments for the BAliBASE set also had surprisingly low fractions of correctly-aligned positions (Tables 4,5,6,7), although the shift errors in the alignments for this method were among the smallest (Tables 8,9,10) and the scores on the low sequence-identity ion family were also consistently high-ranking (Tables 4,5,6,8,9,10). This low performance of the profile-profile methods may reflect greater deviations in the two profiles than in the sequences themselves making them more difficult to align. Since the selection of sequence homologues appears to be an important parameter [30], in future work we plan to analyze the influence of the database search parameters on the accuracy of the different alignment methods, and to test programs such as SHRIMP [70], HMMER3 [71], and HHblits [72] instead of PSI-BLAST.

This study focuses on α-helical membrane protein sequences, so that we obtain gap penalties that are optimal for long helices and are not biased by the inclusion of short β-stranded regions [30]. Optimization against β-barrel proteins is likely to lead to different gap penalty sets, and may result in methods that are particularly useful for that membrane protein architecture.

A concern about the current study is the fact that no structural informational was available to aid with the alignments when the BAliBASE reference set 7 was constructed, and therefore it is possible that these alignments contain errors whose effect we cannot yet know [63]. Nevertheless, the relatively consistent ranking of the different methods on both the BAliBASE and HOMEP2 sets, i.e., with AlignMePS, MSAProbs and HMAP frequently high-ranking, and the profile-profile methods ranked towards the bottom, suggests that our findings are reasonably robust.

As the size of the database of membrane protein structures grows, further assessment of the various methods will be useful. Nevertheless, the results presented here suggest that there is potential for using the specific properties of membrane proteins for training and design in a way that aids the alignment of their sequences.

## Supporting Information

File S1 Contains Figures S1 through S6 and Tables S1 and S2. **Figure S1** Clustering principle used for generation of the HOMEP2 data set. **Figure S2** Correlations between alignment accuracy measures. **Figure S3** Comparison of alignment accuracy when using hydrophobicity scales as input descriptors in AlignMe. **Figure S4** Profiles of the predicted membrane propensity from the three different transmembrane helix prediction methods tested for AlignMe. **Figure S5** Accuracy of HOMEP2 alignments generated by different methods. **Figure S6** Accuracy of homology models built from HOMEP2 alignments generated by different methods. **Table S1** Proteins in the HOMEP2 data set, listed by family **Table S2** Sequence identities between pairs of proteins in the same HOMEP2 family, based on their SKA structural alignments.
(DOCX)

File S2 Dataset of HOMEP2 homologous membrane proteins.
(GZ) Click here for additional data file.

File S3 Manual for AlignMe.
(PDF)

File S4 Source code for AlignMe.
(GZ) Click here for additional data file.
