# Supplementary material for: Alignment of Helical Membrane Protein Sequences Using AlignMe
Source: PLoS One. 2013 Mar 4;8(3):e57731. doi: 10.1371/journal.pone.0057731 (PMC3587630; doi:10.1371/journal.pone.0057731)
Supplement: File S1 — Contains Figures S1 through S6 and Tables S1 and S2. Figure S1 Clustering principle used for generation of the HOMEP2 data set. Figure S2 Correlations between alignment accuracy measures. Figure S3 Comparison of alignment accuracy when using hydrophobicity scales as input descriptors in AlignMe. Figure S4 Profiles of the predicted membrane propensity from the three different transmembrane helix prediction methods tested for AlignMe. Figure S5 Accuracy of HOMEP2 alignments generated by different methods. Figure S6 Accuracy of homology models built from HOMEP2 alignments generated by different methods. Table S1 Proteins in the HOMEP2 data set, listed by family Table S2 Sequence identities between pairs of proteins in the same HOMEP2 family, based on their SKA structural alignments. (DOCX) [file pone.0057731.s001.docx]

**Supplementary Figures and Tables
Stamm et al. PLOS One 2013**


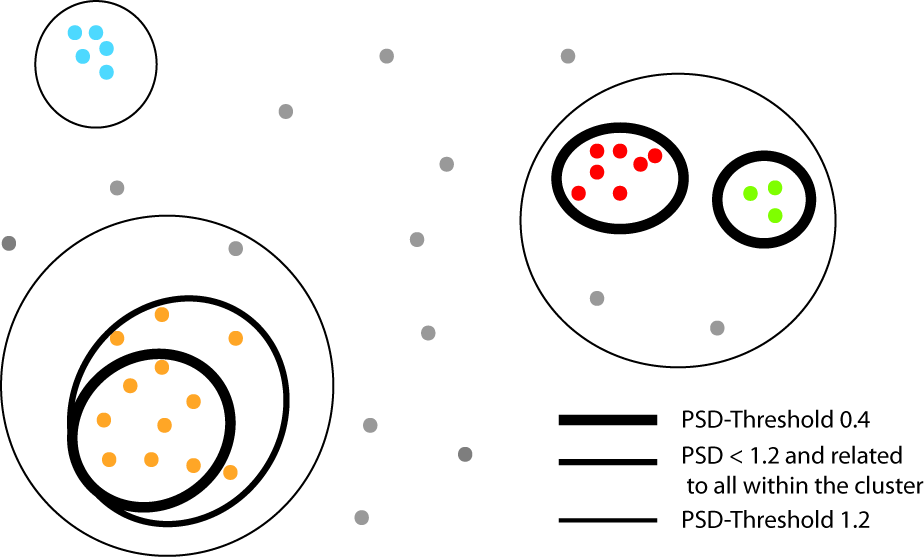


**Figure S1.** Clustering principle used for generation of the HOMEP2 data set*.* Proteins are represented by dots. Dots with the same color are assigned to the same family. Grey dots do not belong to any family. The procedure was as follows. First, all pairs of aligned protein chains with a PSD <1.2 (i.e., within the same SCOP superfamily) were clustered into a common group. Groups in which all protein chains were related to all others within this cut-off were then assigned to be a unique family (blue dots). We then repeated the clustering for all remaining groups, but using a PSD threshold of 0.4, in order to identify proteins belonging to e.g., the same SCOP family, (red and green dots). In some cases, proteins were incorrectly clustered to a given family because small fragments of their structures could be aligned with low PSD values, even though the whole proteins are not structurally related. Therefore, to ensure that all proteins within a cluster are related to one another, any protein chains that SKA was unable to align to all other chains in the family were then excluded from the family. In addition, any protein chains that were related to all members of a family with a PSD value between 0.4 and 1.2 were added to this family (yellow dots). In the final step, pair-wise alignments of all sequences in each family generated using AlignMe (using BLOSUM62, and standard gap opening and extension penalties of 10 and 1, respectively) were used to identify redundant sequences (>85% sequence identity): for two chains of the same protein, only the one with the longest sequence, or the lower total B-factor was retained; for two chains of different proteins, only the one with the higher resolution, or with the smaller R-factor, was retained.


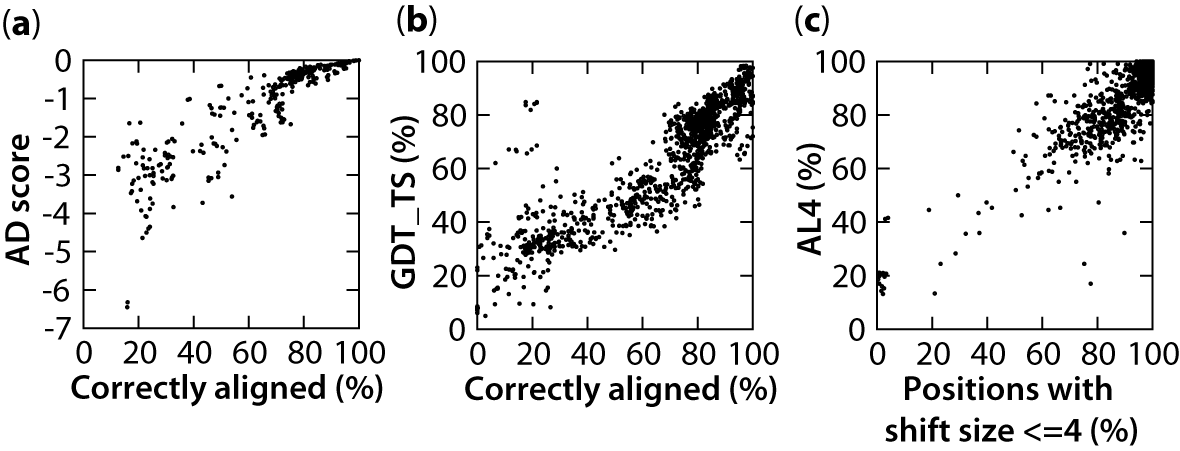


**Figure S2**. Correlations between alignment accuracy measures. All measures were calculated based on HOMEP2 alignments generated with AlignMePST. (**a, b**) Fraction of correctly aligned positions plotted against either the AD score (**a**) or the corresponding model quality measured using GDT_TS (**b**). (**c**) The percentage of residues correctly aligned within a shift of 4 positions is plotted against the corresponding model quality measured using the AL4 score. Correlation coefficients are: (a) 0.90, (b) 0.92 and (c) 0.88.


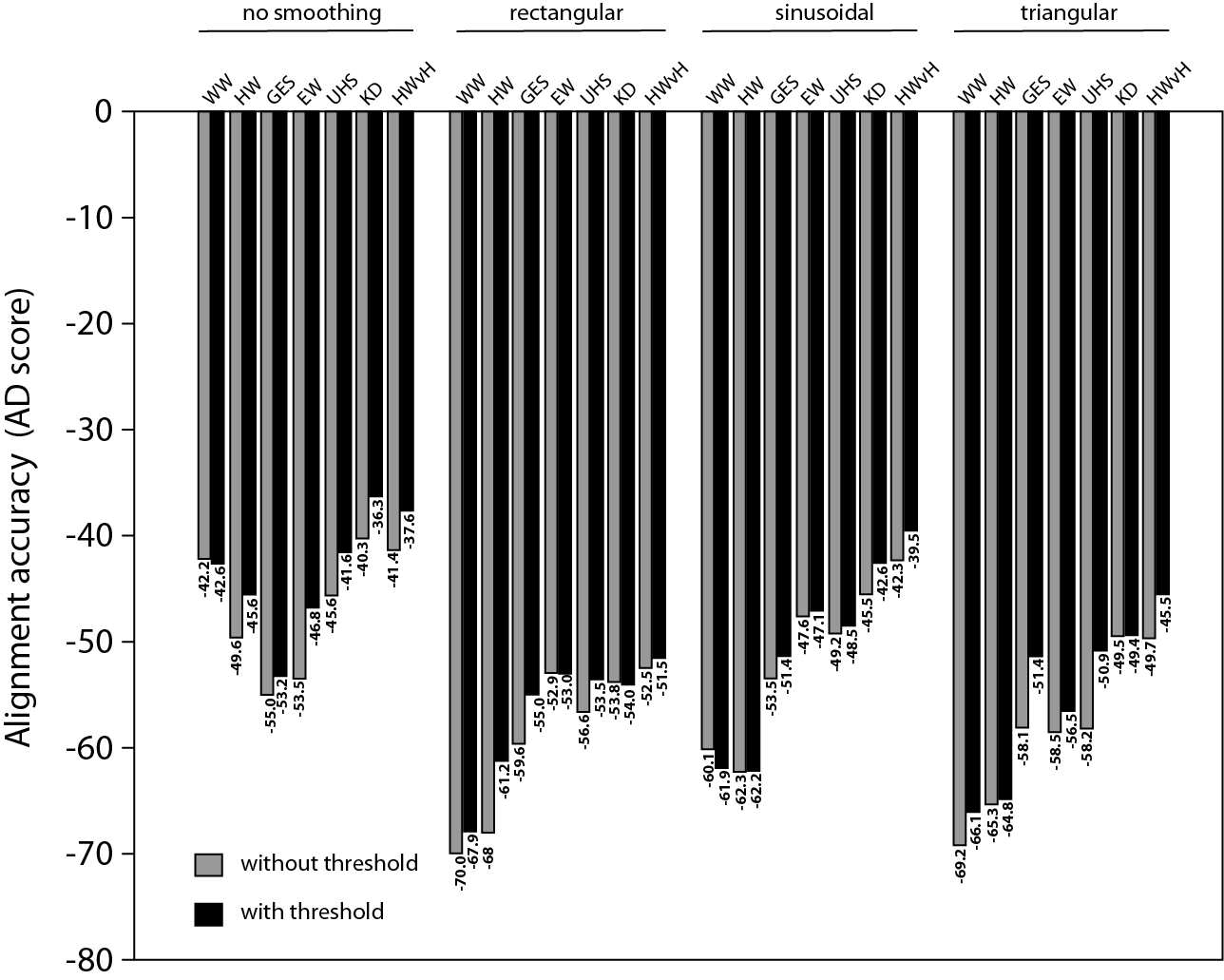


**Figure S3.** Comparison of alignment accuracy when using hydrophobicity scales as input descriptors in AlignMe. The different hydrophobicity scales were either not smoothed (“no smoothing”) as shown also in Figure 1, or window-averaged using a rectangular-, sinusoidal- or triangular-shaped window for averaging. See legend to Figure 1 for more details.


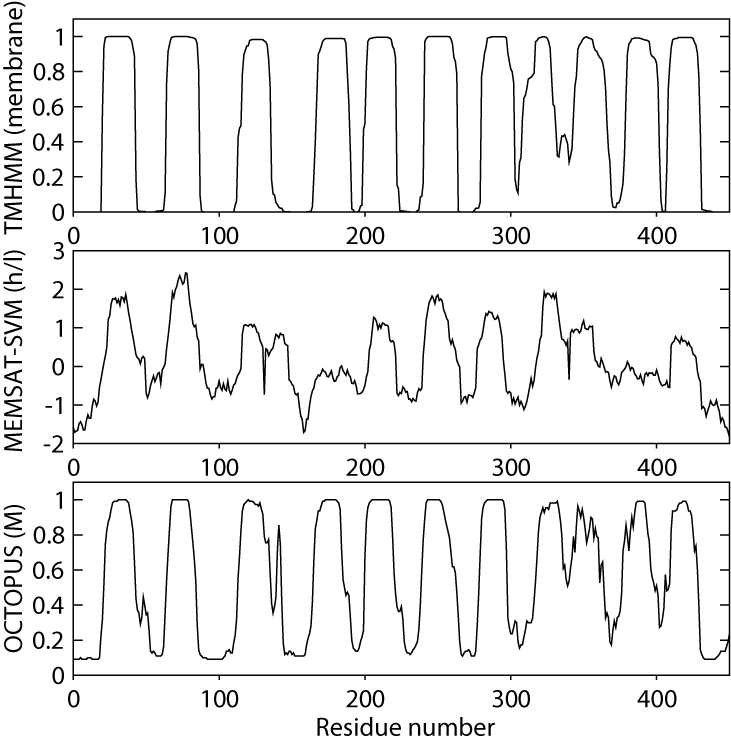


**Figure S4**. Profiles of the predicted membrane propensity from the three different transmembrane helix prediction meth­­ods tested for AlignMe. Predictions were generated for the ClC protein with PDB identifier 1KPL. The *y*-axis label contains the header of the corresponding column in the output from the prediction method.


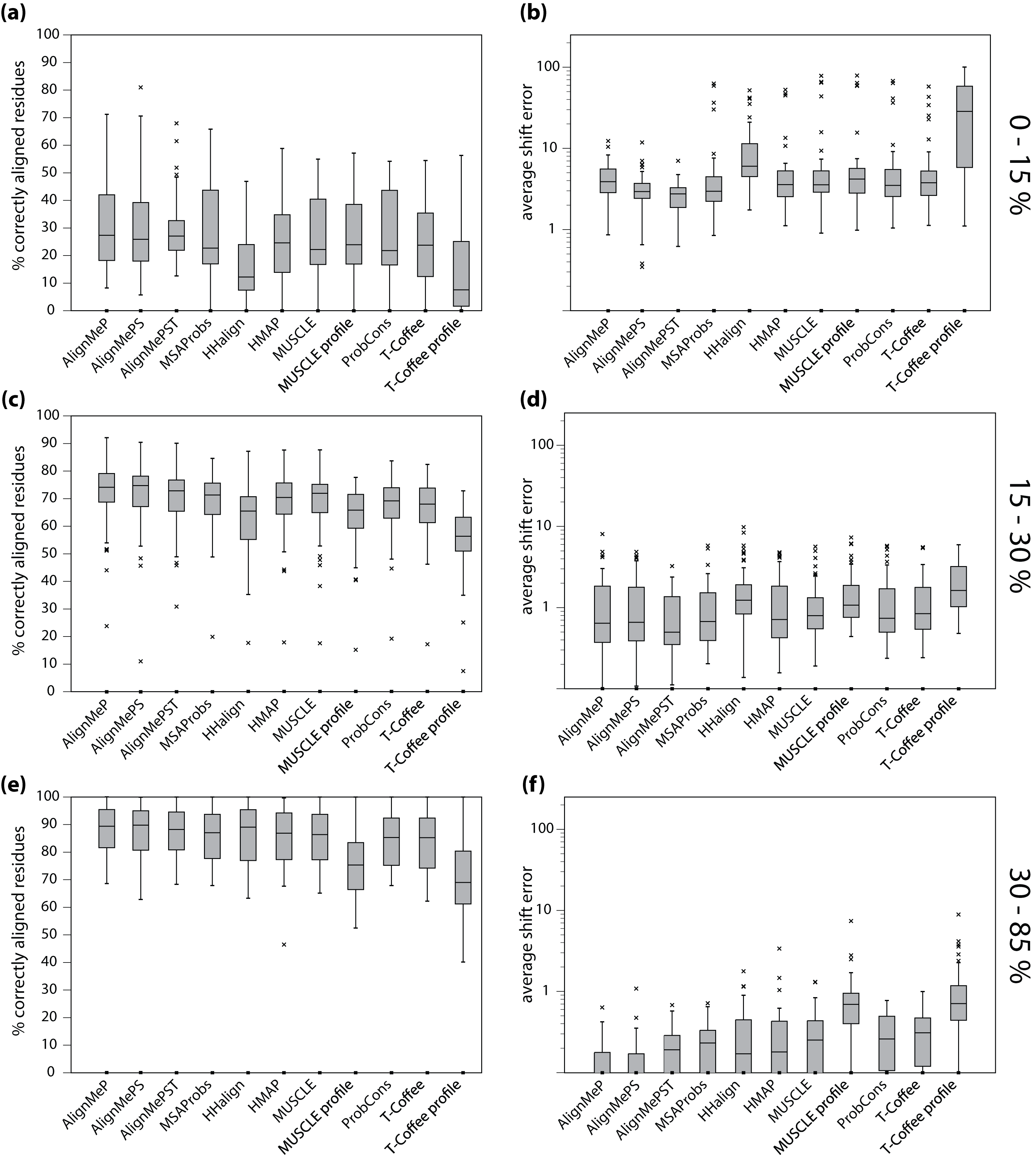


**Figure S5.** Accuracy of HOMEP2 alignments generated by different methods. Accuracy is measured using **(a, c, e)** the percentage of correctly-aligned residues, or **(b, d, f)** the average shift error, shown for all alignments as a box and whisker plot. Alignments are sorted according to the percentage identity between the sequences, namely **(a, b)** <15%, **(c, d)** 15-30% or **(e, f)** >30%.


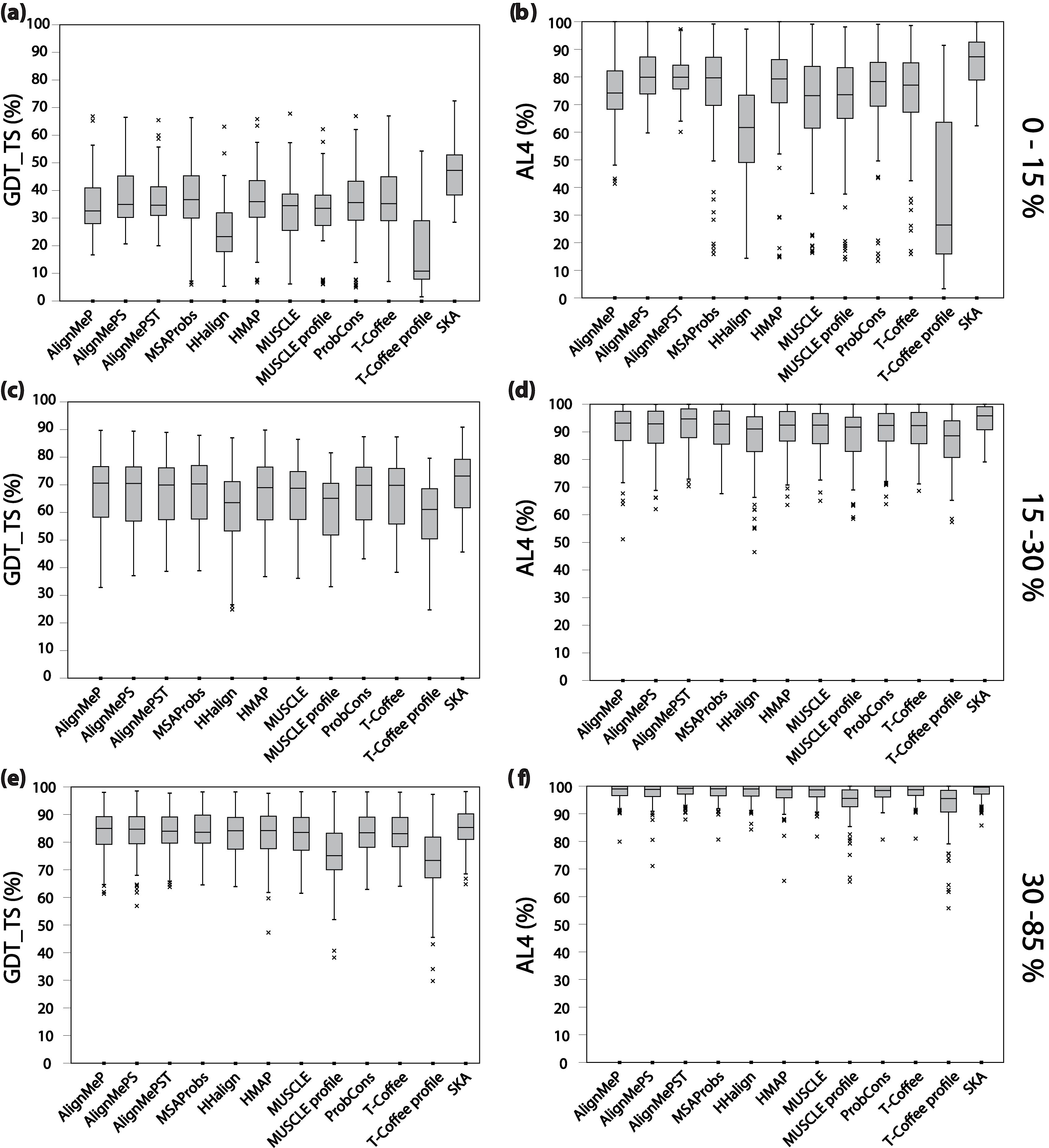


**Figure S6.** Accuracy of homology models built from HOMEP2 alignments generated by different methods. Accuracy is measured using the **(a, c, e)** GDT_TS and **(b, d, f)** AL4 scores of homology models compared to the known structures. Alignments are sorted according to the percentage identity between the sequences, namely **(a, b)** <15%, **(c, d)** 15-30% or **(e, f)** >30%.

**Table S1**. Proteins in the HOMEP2 data set, listed by family

| # | PDB ID | Name | Source | Res  (Å) |
| --- | --- | --- | --- | --- |
| 0 | 2R6G | THE CRYSTAL STRUCTURE OF THE E. COLI MALTOSE TRANSPORTER | *ESCHERICHIA COLI K12* | 2.80 |
| 0 | 3D31 | MODBC FROM METHANOSARCINA ACETIVORANS | *METHANOSARCINA ACETIVORANS* | 3.00 |
| 0 | 2ONK | ABC TRANSPORTER MODBC IN COMPLEX WITH ITS BINDING PROTEIN MODA | *ARCHAEOGLOBUS FULGIDUS* | 3.10 |
| 1 | 2NQ2 | AN INWARD-FACING CONFORMATION OF A PUTATIVE METAL-CHELATE TYPE ABC TRANSPORTER | *HAEMOPHILUS INFLUENZAE* | 2.40 |
| 1 | 2QI9 | ABC-TRANSPORTER BTUCD IN COMPLEX WITH ITS PERIPLASMIC BINDING PROTEIN BTUF | *ESCHERICHIA COLI* | 2.60 |
| 2 | 3B9W | THE 1.3 A RESOLUTION STRUCTURE OF NITROSOMONAS EUROPAEA RH50 AND MECHANISTIC IMPLICATIONS FOR NH3 TRANSPORT BY RHESUS FAMILY PROTEINS | *NITROSOMONAS EUROPAEA* | 1.30 |
| 2 | 1U7G | CRYSTAL STRUCTURE OF AMMONIA CHANNEL AMTB FROM E. COLI | *ESCHERICHIA COLI* | 1.40 |
| 2 | 2B2H | AMMONIUM TRANSPORTER AMT-1 FROM A. FULGIDUS (AS) | *ARCHAEOGLOBUS FULGIDUS* | 1.54 |
| 3 | 2W2E | 1.15 ANGSTROM CRYSTAL STRUCTURE OF P.PASTORIS AQUAPORIN, AQY1, IN A CLOSED CONFORMATION AT PH 3.5 | *PICHIA PASTORIS* | 1.15 |
| 3 | 2F2B | CRYSTAL STRUCTURE OF INTEGRAL MEMBRANE PROTEIN AQUAPORIN AQPM AT 1.68A RESOLUTION | *METHANOTHERMOBACTER MARBURGENSIS STR.* | 1.68 |
| 3 | 3GD8 | CRYSTAL STRUCTURE OF HUMAN AQUAPORIN 4 AT 1.8 AND ITS MECHANISM OF CONDUCTANCE | *HOMO SAPIENS* | 1.80 |
| 3 | 2B6O | ELECTRON CRYSTALLOGRAPHIC STRUCTURE OF LENS AQUAPORIN-0 (AQP0) (LENS MIP) AT 1.9A RESOLUTION, IN A CLOSED PORE STATE | *OVIS ARIES* | 1.90 |
| 3 | 2O9G | CRYSTAL STRUCTURE OF AQPZ MUTANT L170C COMPLEXED WITH MERCURY. | *ESCHERICHIA COLI* | 1.90 |
| 3 | 3D9S | HUMAN AQUAPORIN 5 (AQP5) - HIGH RESOLUTION X-RAY STRUCTURE | *HOMO SAPIENS* | 2.00 |
| 3 | 3LLQ | AQUAPORIN STRUCTURE FROM PLANT PATHOGEN AGROBACTERIUM TUMERFACIENS | *AGROBACTERIUM TUMEFACIENS STR. C58* | 2.01 |
| 3 | 3CN5 | CRYSTAL STRUCTURE OF THE SPINACH AQUAPORIN SOPIP21 S115E, S274E MUTANT | *SPINACIA OLERACEA* | 2.05 |
| 3 | 3C02 | X-RAY STRUCTURE OF THE AQUAGLYCEROPORIN FROM PLASMODIUM FALCIPARUM | *PLASMODIUM FALCIPARUM* | 2.05 |
| 3 | 1LDF | CRYSTAL STRUCTURE OF THE E. COLI GLYCEROL FACILITATOR (GLPF) MUTATION W48F, F200T | *ESCHERICHIA COLI* | 2.10 |
| 3 | 3KLY | PENTAMERIC FORMATE CHANNEL | *VIBRIO CHOLERAE* | 2.10 |
| 3 | 1J4N | CRYSTAL STRUCTURE OF THE AQP1 WATER CHANNEL | *BOS TAURUS* | 2.20 |
| 4 | 1M0L | BACTERIORHODOPSIN/LIPID COMPLEX AT 1.47 A RESOLUTION | *HALOBACTERIUM SALINARUM* | 1.47 |
| 4 | 2JAF | GROUND STATE OF HALORHODOPSIN T203V | *HALOBACTERIUM SALINARIUM* | 1.70 |
| 4 | 1H2S | MOLECULAR BASIS OF TRANSMENBRANE SIGNALLING BY SENSORY RHODOPSIN II-TRANSDUCER COMPLEX | *NATRONOMONAS PHARAONIS* | 1.93 |
| 4 | 1XIO | ANABAENA SENSORY RHODOPSIN | *NOSTOC SP. PCC 7120* | 2.00 |
| 4 | 3A7K | CRYSTAL STRUCTURE OF HALORHODOPSIN FROM NATRONOMONAS PHARAONIS | *NATRONOMONAS PHARAONIS DSM 2160* | 2.00 |
| 4 | 2EI4 | TRIMERIC COMPLEX OF ARCHAERHODOPSIN-2 | *HALOBACTERIUM SP. AUS-2* | 2.10 |
| 5 | 1OTS | STRUCTURE OF THE ESCHERICHIA COLI CLC CHLORIDE CHANNEL AND FAB COMPLEX | *ESCHERICHIA COLI* | 2.51 |
| 5 | 1KPL | CRYSTAL STRUCTURE OF THE CLC CHLORIDE CHANNEL FROM S. TYPHIMURIUM | *SALMONELLA TYPHIMURIUM* | 3.00 |
| 6 | 2ZT9 | CRYSTAL STRUCTURE OF THE CYTOCHROME B6F COMPLEX FROM NOSTOC SP. PCC 7120 | *NOSTOC SP. PCC 7120* | 3.00 |
| 6 | 1Q90 | STRUCTURE OF THE CYTOCHROME B6F (PLASTOHYDROQUINONE : PLASTOCYANIN OXIDOREDUCTASE) FROM CHLAMYDOMONAS REINHARDTII | *CHLAMYDOMONAS REINHARDTII* | 3.10 |
| 7 | 3CX5 | STRUCTURE OF COMPLEX III WITH BOUND CYTOCHROME C IN REDUCED STATE AND DEFINITION OF A MINIMAL CORE INTERFACE FOR ELECTRON TRANSFER. | *SACCHAROMYCES CEREVISIAE* | 1.90 |
| 7 | 2A06 | BOVINE CYTOCHROME BC1 COMPLEX WITH STIGMATELLIN BOUND | *BOS TAURUS* | 2.10 |
| 7 | 2QJY | CRYSTAL STRUCTURE OF RHODOBACTER SPHAEROIDES DOUBLE MUTANT WITH STIGMATELLIN AND UQ2 | *RHODOBACTER SPHAEROIDES* | 2.40 |
| 7 | 3L70 | CYTOCHROME BC1 COMPLEX FROM CHICKEN WITH TRIFLOXYSTROBIN BOUND | *GALLUS GALLUS* | 2.75 |
| 8 | 1V54 | BOVINE HEART CYTOCHROME C OXIDASE AT THE FULLY OXIDIZED STATE | *BOS TAURUS* | 1.80 |
| 8 | 2GSM | CATALYTIC CORE (SUBUNITS I AND II) OF CYTOCHROME C OXIDASE FROM RHODOBACTER SPHAEROIDES | *RHODOBACTER SPHAEROIDES* | 2.00 |
| 8 | 3HB3 | HIGH RESOLUTION CRYSTAL STRUCTURE OF PARACOCCUS DENITRIFICANS CYTOCHROME C OXIDASE | *PARACOCCUS DENITRIFICANS* | 2.25 |
| 9 | 1V54 | BOVINE HEART CYTOCHROME C OXIDASE AT THE FULLY OXIDIZED STATE | *BOS TAURUS* | 1.80 |
| 9 | 1M56 | STRUCTURE OF CYTOCHROME C OXIDASE FROM RHODOBACTOR SPHAEROIDES (WILD TYPE) | *RHODOBACTER SPHAEROIDES* | 2.30 |
| 9 | 1QLE | CRYO-STRUCTURE OF THE PARACOCCUS DENITRIFICANS FOUR-SUBUNIT CYTOCHROME C OXIDASE IN THE COMPLETELY OXIDIZED STATE COMPLEXED WITH AN ANTIBODY FV FRAGMENT | *PARACOCCUS DENITRIFICANS* | 3.0 |
| 10 | 2DYR | BOVINE HEART CYTOCHROME C OXIDASE AT THE FULLY OXIDIZED STATE | *BOS TAURUS* | 1.80 |
| 10 | 2GSM | CATALYTIC CORE (SUBUNITS I AND II) OF CYTOCHROME C OXIDASE FROM RHODOBACTER SPHAEROIDES | *RHODOBACTER SPHAEROIDES* | 2.00 |
| 10 | 3HB3 | HIGH RESOLUTION CRYSTAL STRUCTURE OF PARACOCCUS DENITRIFICANS CYTOCHROME C OXIDASE | *PARACOCCUS DENITRIFICANS* | 2.25 |
| 11 | 3B44 | CRYSTAL STRUCTURE OF GLPG W136A MUTANT | *ESCHERICHIA COLI* | 1.70 |
| 11 | 2NR9 | CRYSTAL STRUCTURE OF GLPG, RHOMBOID PEPTIDASE FROM HAEMOPHILUS INFLUENZAE | *HAEMOPHILUS INFLUENZAE 86-028NP* | 2.20 |
| 12 | 2RH1 | HIGH RESOLUTION CRYSTAL STRUCTURE OF HUMAN B2-ADRENERGIC G PROTEIN-COUPLED RECEPTOR. | *HOMO SAPIENS, ENTEROBACTERIA PHAGE T4* | 2.40 |
| 12 | 2Z73 | CRYSTAL STRUCTURE OF SQUID RHODOPSIN | *TODARODES PACIFICUS* | 2.50 |
| 12 | 3KJ6 | CRYSTAL STRUCTURE OF A METHYLATED BETA2 ADRENERGIC RECEPTOR- FAB COMPLEX | *HOMO SAPIENS* | 3.40 |
| 12 | 2VT4 | TURKEY BETA1 ADRENERGIC RECEPTOR WITH STABILISING MUTATIONS AND BOUND CYANOPINDOLOL | *MELEAGRIS GALLOPAVO* | 2.7 |
| 13 | 2CFQ | SUGAR FREE LACTOSE PERMEASE AT NEUTRAL PH | *ESCHERICHIA COLI* | 2.95 |
| 13 | 1PW4 | CRYSTAL STRUCTURE OF THE GLYCEROL-3-PHOSPHATE TRANSPORTER FROM E.COLI | *ESCHERICHIA COLI* | 3.30 |
| 14 | 2J8S | DRUG EXPORT PATHWAY OF MULTIDRUG EXPORTER ACRB REVEALED BY DARPIN INHIBITORS | *ESCHERICHIA COLI* | 2.54 |
| 14 | 2V50 | THE MISSING PART OF THE BACTERIAL MEXAB-OPRM SYSTEM: STRUCTURAL DETERMINATION OF THE MULTIDRUG EXPORTER MEXB | *PSEUDOMONAS AERUGINOSA PA01* | 3.00 |
| 15 | 3EAM | AN OPEN-PORE STRUCTURE OF A BACTERIAL PENTAMERIC LIGAND- GATED ION CHANNEL | *GLOEOBACTER VIOLACEUS* | 2.90 |
| 15 | 2VL0 | X-RAY STRUCTURE OF A PENTAMERIC LIGAND GATED ION CHANNEL FROM ERWINIA CHRYSANTHEMI (ELIC) | *ERWINIA CHRYSANTHEMI* | 3.3 |
| 16 | 1JB0 | CRYSTAL STRUCTURE OF PHOTOSYSTEM I: A PHOTOSYNTHETIC REACTION CENTER AND CORE ANTENNA SYSTEM FROM CYANOBACTERIA | *SYNECHOCOCCUS ELONGATUS* | 2.50 |
| 16 | 2WSC | IMPROVED MODEL OF PLANT PHOTOSYSTEM I | *PISUM SATIVUM* | 3.30 |
| 17 | 1RZH | PHOTOSYNTHETIC REACTION CENTER DOUBLE MUTANT FROM RHODOBACTER SPHAEROIDES WITH ASP L213 REPLACED WITH ASN AND ARG M233 REPLACED WITH CYS IN THE CHARGE-NEUTRAL DQAQB STATE (TRIGONAL FORM) | *RHODOBACTER SPHAEROIDES* | 1.80 |
| 17 | 1RZH | PHOTOSYNTHETIC REACTION CENTER DOUBLE MUTANT FROM RHODOBACTER SPHAEROIDES WITH ASP L213 REPLACED WITH ASN AND ARG M233 REPLACED WITH CYS IN THE CHARGE-NEUTRAL DQAQB STATE (TRIGONAL FORM) | *RHODOBACTER SPHAEROIDES* | 1.80 |
| 17 | 2WJN | LIPIDIC SPONGE PHASE CRYSTAL STRUCTURE OF PHOTOSYNTHETIC REACTION CENTRE FROM BLASTOCHLORIS VIRIDIS (HIGH DOSE) | *RHODOPSEUDOMONAS VIRIDIS* | 1.86 |
| 17 | 2WJN | LIPIDIC SPONGE PHASE CRYSTAL STRUCTURE OF PHOTOSYNTHETIC REACTION CENTRE FROM BLASTOCHLORIS VIRIDIS (HIGH DOSE) | *RHODOPSEUDOMONAS VIRIDIS* | 1.86 |
| 17 | 1EYS | CRYSTAL STRUCTURE OF PHOTOSYNTHETIC REACTION CENTER FROM A THERMOPHILIC BACTERIUM, THERMOCHROMATIUM TEPIDUM | *THERMOCHROMATIUM TEPIDUM* | 2.20 |
| 17 | 1EYS | CRYSTAL STRUCTURE OF PHOTOSYNTHETIC REACTION CENTER FROM A THERMOPHILIC BACTERIUM, THERMOCHROMATIUM TEPIDUM | *THERMOCHROMATIUM TEPIDUM* | 2.20 |
| 17 | 3BZ1 | CRYSTAL STRUCTURE OF CYANOBACTERIAL PHOTOSYSTEM II (PART 1 OF 2). THIS FILE CONTAINS FIRST MONOMER OF PSII DIMER | *THERMOSYNECHOCOCCUS ELONGATUS* | 2.90 |
| 17 | 3BZ1 | CRYSTAL STRUCTURE OF CYANOBACTERIAL PHOTOSYSTEM II (PART 1 OF 2). THIS FILE CONTAINS FIRST MONOMER OF PSII DIMER | *THERMOSYNECHOCOCCUS ELONGATUS* | 2.90 |
| 18 | 1RH5 | THE STRUCTURE OF A PROTEIN CONDUCTING CHANNEL | *METHANOCALDOCOCCUS JANNASCHII* | 3.20 |
| 18 | 2ZJS | CRYSTAL STRUCTURE OF SECYE TRANSLOCON FROM THERMUS THERMOPHILUS WITH A FAB FRAGMENT | *THERMUS THERMOPHILUS* | 3.20 |
| 19 | 2H88 | AVIAN MITOCHONDRIAL RESPIRATORY COMPLEX II AT 1.8 ANGSTROM RESOLUTION | *GALLUS GALLUS* | 1.74 |
| 19 | 2H88 | AVIAN MITOCHONDRIAL RESPIRATORY COMPLEX II AT 1.8 ANGSTROM RESOLUTION | *GALLUS GALLUS* | 1.74 |
| 19 | 1ZOY | CRYSTAL STRUCTURE OF MITOCHONDRIAL RESPIRATORY COMPLEX II FROM PORCINE HEART AT 2.4 ANGSTROMS | *SUS SCROFA* | 2.40 |
| 19 | 2WDQ | E. COLI SUCCINATE:QUINONE OXIDOREDUCTASE (SQR) WITH CARBOXIN BOUND | *ESCHERICHIA COLI* | 2.40 |
| 19 | 1ZOY | CRYSTAL STRUCTURE OF MITOCHONDRIAL RESPIRATORY COMPLEX II FROM PORCINE HEART AT 2.4 ANGSTROMS | *SUS SCROFA* | 2.40 |
| 19 | 2WDQ | E. COLI SUCCINATE:QUINONE OXIDOREDUCTASE (SQR) WITH CARBOXIN BOUND | *ESCHERICHIA COLI* | 2.40 |
| 19 | 1KF6 | E. COLI QUINOL-FUMARATE REDUCTASE WITH BOUND INHIBITOR HQNO | *ESCHERICHIA COLI* | 2.70 |
| 20 | 2A65 | CRYSTAL STRUCTURE OF LEUTAA, A BACTERIAL HOMOLOG OF NA+/CL-- DEPENDENT NEUROTRANSMITTER TRANSPORTERS | *AQUIFEX AEOLICUS VF5* | 1.65 |
| 20 | 3GIA | CRYSTAL STRUCTURE OF APCT TRANSPORTER | *METHANOCALDOCOCCUS JANNASCHII* | 2.32 |
| 20 | 2JLN | STRUCTURE OF MHP1, A NUCLEOBASE-CATION-SYMPORT-1 FAMILY TRANSPORTER | *MICROBACTERIUM LIQUEFACIENS* | 2.85 |
| 20 | 3L1L | STRUCTURE OF ARG-BOUND ESCHERICHIA COLI ADIC | *ESCHERICHIA COLI* | 3.00 |
| 20 | 2WIT | CRYSTAL STRUCTURE OF THE SODIUM-COUPLED GLYCINE BETAINE SYMPORTER BETP FROM CORYNEBACTERIUM GLUTAMICUM WITH BOUND SUBSTRATE | *CORYNEBACTERIUM GLUTAMICUM* | 3.35 |
| 21 | 1WPG | CRYSTAL STRUCTURE OF THE SR CA2+-ATPASE WITH MGF4 | *ORYCTOLAGUS CUNICULUS* | 2.30 |
| 21 | 2ZXE | CRYSTAL STRUCTURE OF THE SODIUM - POTASSIUM PUMP IN THE E2.2K+.PI STATE | *SQUALUS ACANTHIAS* | 2.40 |

The numbers in the first column refer to the following families: (0) ABC transporters - efflux, (1) ABC transporters - influx, (2) Ammonium transporters, (3) Aquaporins, (4) Bacterial rhodopsins, (5) ClC transporters, (6) Cytochrome b6f, (7) Cytochrome bc1, (8) Cytochrome c oxidases - 2TM-helix subunit, (9) Cytochrome c oxidases - 7TM-helix subunit, (10) Cytochrome c oxidases - 12TM-helix subunit, (11) GlpG, (12) GPCR, (13) MFS transporters, (14) Multi-drug exporters, (15) Pentameric ion channels, (16) Photosystem I, (17) Photosystem II, (18) Protein conducting channels, (19) Reductases, (20) LeuT-fold transporters, (21) Sodium/potassium pumps.

**Table S2.** Sequence identities between pairs of proteins in the same HOMEP2 family, based on their SKA structural alignments

| Family | PDB1 | Chain ID from PDB1 | PDB2 | Chain ID from PDB2 | PSD value | % sequence identity |
| --- | --- | --- | --- | --- | --- | --- |
| 0 | 3D31 | C | 2R6G | G | 0.47 | 19.5 |
| 0 | 2ONK | C | 2R6G | G | 0.50 | 20.5 |
| 0 | 3D31 | C | 2ONK | C | 0.07 | 50.8 |
| 1 | 2NQ2 | A | 2QI9 | B | 0.34 | 31.9 |
| 2 | 1U7G | A | 3B9W | A | 0.26 | 18.3 |
| 2 | 3B9W | A | 2B2H | A | 0.28 | 18.6 |
| 2 | 1U7G | A | 2B2H | A | 0.13 | 34.1 |
| 3 | 2O9G | A | 3KLY | C | 0.57 | 7.3 |
| 3 | 1J4N | A | 3KLY | C | 0.58 | 8.3 |
| 3 | 3CN5 | A | 3KLY | C | 0.50 | 9.3 |
| 3 | 1LDF | A | 3KLY | C | 0.52 | 9.7 |
| 3 | 2B6O | A | 3KLY | C | 0.57 | 9.7 |
| 3 | 2F2B | A | 3KLY | C | 0.38 | 9.9 |
| 3 | 3LLQ | A | 3KLY | C | 0.47 | 9.9 |
| 3 | 3C02 | A | 3KLY | C | 0.47 | 10.4 |
| 3 | 3GD8 | A | 3KLY | C | 0.52 | 10.4 |
| 3 | 2W2E | A | 3KLY | C | 0.44 | 11.1 |
| 3 | 3D9S | D | 3KLY | C | 0.47 | 13.1 |
| 3 | 2W2E | A | 3C02 | A | 0.14 | 17.8 |
| 3 | 3CN5 | A | 3C02 | A | 0.22 | 20.5 |
| 3 | 3GD8 | A | 3C02 | A | 0.13 | 21.9 |
| 3 | 2W2E | A | 1LDF | A | 0.15 | 22.4 |
| 3 | 1J4N | A | 3C02 | A | 0.17 | 23.2 |
| 3 | 2W2E | A | 2F2B | A | 0.11 | 23.2 |
| 3 | 3C02 | A | 2O9G | A | 0.12 | 23.6 |
| 3 | 2W2E | A | 2O9G | A | 0.13 | 23.8 |
| 3 | 2B6O | A | 2W2E | A | 0.11 | 24.1 |
| 3 | 3C02 | A | 3D9S | D | 0.15 | 24.2 |
| 3 | 2B6O | A | 3C02 | A | 0.16 | 24.3 |
| 3 | 3LLQ | A | 3C02 | A | 0.10 | 24.3 |
| 3 | 3GD8 | A | 1LDF | A | 0.13 | 24.7 |
| 3 | 2W2E | A | 3LLQ | A | 0.12 | 24.8 |
| 3 | 2W2E | A | 3D9S | D | 0.19 | 25.2 |
| 3 | 3CN5 | A | 1LDF | A | 0.16 | 25.3 |
| 3 | 1J4N | A | 1LDF | A | 0.14 | 25.4 |
| 3 | 2B6O | A | 1LDF | A | 0.17 | 25.4 |
| 3 | 3CN5 | A | 2O9G | A | 0.13 | 26.4 |
| 3 | 1J4N | A | 2F2B | A | 0.17 | 26.7 |
| 3 | 3D9S | D | 1LDF | A | 0.13 | 26.9 |
| 3 | 2B6O | A | 2F2B | A | 0.09 | 27.1 |
| 3 | 2W2E | A | 3GD8 | A | 0.13 | 27.2 |
| 3 | 3LLQ | A | 1LDF | A | 0.15 | 27.8 |
| 3 | 2W2E | A | 1J4N | A | 0.18 | 28.1 |
| 3 | 3CN5 | A | 2F2B | A | 0.17 | 28.2 |
| 3 | 2O9G | A | 1LDF | A | 0.15 | 28.3 |
| 3 | 2W2E | A | 3CN5 | A | 0.18 | 28.4 |
| 3 | 3GD8 | A | 2O9G | A | 0.15 | 28.5 |
| 3 | 3C02 | A | 2F2B | A | 0.15 | 28.7 |
| 3 | 3LLQ | A | 3CN5 | A | 0.08 | 28.8 |
| 3 | 3GD8 | A | 3LLQ | A | 0.12 | 29.7 |
| 3 | 2B6O | A | 3CN5 | A | 0.09 | 30.5 |
| 3 | 2B6O | A | 3LLQ | A | 0.12 | 31.1 |
| 3 | 1J4N | A | 2O9G | A | 0.16 | 31.5 |
| 3 | 3LLQ | A | 1J4N | A | 0.12 | 31.9 |
| 3 | 1LDF | A | 2F2B | A | 0.14 | 32.1 |
| 3 | 2O9G | A | 2F2B | A | 0.14 | 32.6 |
| 3 | 3LLQ | A | 2F2B | A | 0.14 | 32.6 |
| 3 | 3C02 | A | 1LDF | A | 0.11 | 32.7 |
| 3 | 3GD8 | A | 2F2B | A | 0.10 | 32.9 |
| 3 | 3D9S | D | 2F2B | A | 0.12 | 33.1 |
| 3 | 3CN5 | A | 3D9S | D | 0.11 | 33.6 |
| 3 | 2B6O | A | 2O9G | A | 0.15 | 33.8 |
| 3 | 3LLQ | A | 3D9S | D | 0.13 | 33.8 |
| 3 | 2O9G | A | 3D9S | D | 0.14 | 33.9 |
| 3 | 3GD8 | A | 3CN5 | A | 0.08 | 37.0 |
| 3 | 1J4N | A | 3CN5 | A | 0.09 | 38.7 |
| 3 | 1J4N | A | 3D9S | D | 0.13 | 45.3 |
| 3 | 2B6O | A | 3GD8 | A | 0.04 | 45.4 |
| 3 | 3GD8 | A | 1J4N | A | 0.06 | 45.8 |
| 3 | 2B6O | A | 1J4N | A | 0.07 | 47.1 |
| 3 | 3GD8 | A | 3D9S | D | 0.05 | 51.7 |
| 3 | 2B6O | A | 3D9S | D | 0.09 | 56.2 |
| 3 | 3LLQ | A | 2O9G | A | 0.02 | 72.5 |
| 4 | 1XIO | A | 3A7K | A | 0.14 | 20.2 |
| 4 | 1H2S | A | 3A7K | A | 0.10 | 21.5 |
| 4 | 2JAF | A | 1XIO | A | 0.13 | 24.4 |
| 4 | 1H2S | A | 2JAF | A | 0.13 | 25.3 |
| 4 | 1XIO | A | 1M0L | A | 0.15 | 27.3 |
| 4 | 1H2S | A | 1M0L | A | 0.09 | 27.7 |
| 4 | 1H2S | A | 1XIO | A | 0.11 | 28.5 |
| 4 | 1M0L | A | 3A7K | A | 0.07 | 29.1 |
| 4 | 2EI4 | A | 1H2S | A | 0.09 | 29.8 |
| 4 | 2EI4 | A | 3A7K | A | 0.11 | 30.2 |
| 4 | 2EI4 | A | 1XIO | A | 0.17 | 31.2 |
| 4 | 2EI4 | A | 2JAF | A | 0.10 | 31.2 |
| 4 | 2JAF | A | 1M0L | A | 0.09 | 31.5 |
| 4 | 2EI4 | A | 1M0L | A | 0.02 | 55.2 |
| 4 | 2JAF | A | 3A7K | A | 0.04 | 57.1 |
| 5 | 1KPL | B | 1OTS | A | 0.04 | 76.6 |
| 6 | 1Q90 | D | 2ZT9 | B | 0.03 | 76.6 |
| 7 | 2QJY | A | 3CX5 | N | 0.09 | 45.0 |
| 7 | 2QJY | A | 3L70 | C | 0.09 | 45.3 |
| 7 | 2A06 | P | 2QJY | A | 0.06 | 46.4 |
| 7 | 2A06 | P | 3CX5 | N | 0.03 | 50.3 |
| 7 | 3CX5 | N | 3L70 | C | 0.03 | 51.2 |
| 7 | 2A06 | P | 3L70 | C | 0.02 | 73.6 |
| 8 | 1V54 | B | 3HB3 | B | 0.07 | 32.6 |
| 8 | 1V54 | B | 2GSM | B | 0.07 | 34.0 |
| 8 | 3HB3 | B | 2GSM | B | 0.03 | 57.1 |
| 9 | 1V54 | C | 1M56 | C | 0.04 | 48.1 |
| 9 | 1V54 | C | 1QLE | C | 0.05 | 49.6 |
| 9 | 1QLE | C | 1M56 | C | 0.06 | 70.6 |
| 10 | 2DYR | A | 2GSM | A | 0.04 | 51.5 |
| 10 | 2DYR | A | 3HB3 | A | 0.04 | 52.4 |
| 10 | 2GSM | A | 3HB3 | A | 0.02 | 82.3 |
| 11 | 3B44 | A | 2NR9 | A | 0.09 | 36.0 |
| 12 | 2Z73 | A | 3KJ6 | A | 0.59 | 6.3 |
| 12 | 2Z73 | A | 2RH1 | A | 0.48 | 18.0 |
| 12 | 2Z73 | A | 2VT4 | A | 0.35 | 20.5 |
| 12 | 3KJ6 | A | 2RH1 | A | 0.52 | 24.6 |
| 12 | 2VT4 | A | 3KJ6 | A | 0.24 | 54.4 |
| 12 | 2VT4 | A | 2RH1 | A | 0.11 | 61.5 |
| 13 | 1PW4 | A | 2CFQ | A | 0.72 | 8.7 |
| 14 | 2J8S | A | 2V50 | B | 0.36 | 68.5 |
| 15 | 2VL0 | D | 3EAM | B | 0.29 | 21.7 |
| 16 | 2WSC | B | 1JB0 | B | 0.39 | 74.3 |
| 17 | 3BZ1 | D | 1EYS | M | 0.66 | 12.5 |
| 17 | 2WJN | M | 3BZ1 | A | 0.37 | 13.1 |
| 17 | 1EYS | L | 3BZ1 | A | 0.42 | 15.0 |
| 17 | 1EYS | M | 3BZ1 | A | 0.52 | 15.0 |
| 17 | 3BZ1 | D | 1RZH | M | 0.59 | 15.0 |
| 17 | 3BZ1 | D | 2WJN | M | 0.43 | 15.1 |
| 17 | 1RZH | M | 3BZ1 | A | 0.42 | 15.7 |
| 17 | 3BZ1 | D | 1EYS | L | 0.39 | 15.8 |
| 17 | 2WJN | L | 3BZ1 | A | 0.36 | 16.1 |
| 17 | 3BZ1 | D | 1RZH | L | 0.41 | 16.1 |
| 17 | 3BZ1 | D | 2WJN | L | 0.33 | 16.3 |
| 17 | 1RZH | L | 3BZ1 | A | 0.44 | 16.6 |
| 17 | 2WJN | L | 2WJN | M | 0.19 | 23.2 |
| 17 | 1EYS | L | 2WJN | M | 0.21 | 23.5 |
| 17 | 1RZH | L | 2WJN | M | 0.19 | 25.5 |
| 17 | 1EYS | L | 1EYS | M | 0.24 | 25.8 |
| 17 | 1RZH | L | 1EYS | M | 0.23 | 26.7 |
| 17 | 2WJN | L | 1EYS | M | 0.18 | 26.7 |
| 17 | 1RZH | M | 1RZH | L | 0.22 | 27.5 |
| 17 | 1EYS | L | 1RZH | M | 0.24 | 28.6 |
| 17 | 2WJN | L | 1RZH | M | 0.22 | 29.3 |
| 17 | 3BZ1 | D | 3BZ1 | A | 0.11 | 29.6 |
| 17 | 1RZH | M | 2WJN | M | 0.06 | 46.8 |
| 17 | 2WJN | L | 1RZH | L | 0.02 | 58.1 |
| 17 | 2WJN | M | 1EYS | M | 0.04 | 59.0 |
| 17 | 1RZH | M | 1EYS | M | 0.05 | 59.5 |
| 17 | 2WJN | L | 1EYS | L | 0.03 | 65.1 |
| 17 | 1EYS | L | 1RZH | L | 0.02 | 65.6 |
| 18 | 1RH5 | A | 2ZJS | Y | 0.58 | 15.8 |
| 19 | 2H88 | C | 2H88 | D | 0.65 | 6.7 |
| 19 | 1KF6 | D | 2H88 | C | 0.77 | 7.0 |
| 19 | 1KF6 | D | 1ZOY | D | 0.52 | 7.2 |
| 19 | 2WDQ | K | 2H88 | D | 0.11 | 7.2 |
| 19 | 2WDQ | L | 2H88 | C | 0.87 | 7.4 |
| 19 | 1KF6 | D | 1ZOY | C | 0.94 | 7.8 |
| 19 | 1ZOY | C | 1ZOY | D | 0.58 | 8.3 |
| 19 | 1ZOY | C | 2H88 | D | 0.54 | 8.4 |
| 19 | 1KF6 | D | 2H88 | D | 0.56 | 9.1 |
| 19 | 2WDQ | K | 1ZOY | D | 0.09 | 9.9 |
| 19 | 1KF6 | D | 2WDQ | K | 0.46 | 10.0 |
| 19 | 1ZOY | D | 2H88 | C | 0.47 | 10.0 |
| 19 | 2WDQ | L | 1ZOY | C | 0.89 | 10.7 |
| 19 | 2WDQ | L | 1ZOY | D | 0.19 | 11.6 |
| 19 | 2WDQ | L | 2H88 | D | 0.21 | 11.7 |
| 19 | 1KF6 | D | 2WDQ | L | 0.38 | 14.3 |
| 19 | 2WDQ | K | 2H88 | C | 0.43 | 14.6 |
| 19 | 2WDQ | K | 2WDQ | L | 0.60 | 15.9 |
| 19 | 2WDQ | K | 1ZOY | C | 0.33 | 18.5 |
| 19 | 1ZOY | C | 2H88 | C | 0.03 | 70.0 |
| 19 | 1ZOY | D | 2H88 | D | 0.01 | 76.8 |
| 20 | 2WIT | A | 2JLN | A | 1.06 | 5.5 |
| 20 | 2A65 | A | 2JLN | A | 1.03 | 6.6 |
| 20 | 3GIA | A | 2JLN | A | 0.91 | 7.1 |
| 20 | 2WIT | A | 3GIA | A | 0.88 | 7.8 |
| 20 | 2WIT | A | 2A65 | A | 0.96 | 8.0 |
| 20 | 2A65 | A | 3GIA | A | 1.07 | 8.1 |
| 20 | 2JLN | A | 3L1L | A | 0.84 | 8.1 |
| 20 | 2WIT | A | 3L1L | A | 0.90 | 8.7 |
| 20 | 2A65 | A | 3L1L | A | 1.19 | 9.4 |
| 20 | 3GIA | A | 3L1L | A | 0.49 | 16.8 |
| 21 | 2ZXE | A | 1WPG | A | 0.65 | 27.9 |

Structural (reference) alignments were generated using SKA between pairs of protein chains, and the PSD value and the percentage sequence identity for the corresponding sequence alignment was computed. Because alignment of structures in 3D cannot always be converted to a sequence alignment unambiguously, a few of the reference alignments were modified manually. In particular, to correct large internal gaps flanked by one or two terminal residues, those terminal residues were moved to the other end of the gap, resulting instead in a long terminal gap. A large cytoplasmic domain inserted for the purposes of enhancing crystallization was removed from the sequence of PDB entry 2RH1. See legend in Table S1 for more details.
