# Supplementary material for: Alignment of Helical Membrane Protein Sequences Using AlignMe
Source: PLoS One. 2013 Mar 4;8(3):e57731. doi: 10.1371/journal.pone.0057731 (PMC3587630; doi:10.1371/journal.pone.0057731)
Supplement: File S3 — Manual for AlignMe. (PDF) [file pone.0057731.s003.pdf]

AlignMe Manual

Version 1.1

Rene Staritzbichler, Marcus Stamm,
Kamil Khafizov and Lucy R. Forrest

Max Planck Institute of Biophysics
Frankfurt am Main
60438 Germany

1) Introduction....3
2) Using AlignMe with optimized parameters....3
3) Required flags....5
  3.1) Pair-wise sequence-to-sequence alignments....6
    a) Similarity Score File for a pair-wise sequence alignment based on similarity matrices: 6
    b) Similarity Score File for a pair-wise sequence alignment based on Position Specific Matrices....7
    c) Similarity Score File for a pair-wise sequence alignment based on scales (e.g. hydrophobicity): 8
    d) Similarity Score File for a pair-wise sequence alignment based on profiles (e.g. secondary structure predictions)....9
    e) Similarity Score File for a pair-wise sequence alignment based on a combination of matrices, scales or profiles....9
    Basic Tutorial for Pair-wise Alignments....10
  3.2) Alignment of two Multiple Sequence Alignments....13
    3.2.1) Similarity Score File for an alignment of two MSAs....13
    3.2.2) Example....14
  3.3) Pair-wise profile-to-profile alignments....15
    3.3.1) Similarity Score File for a pairwise profile-to-profile alignment....15
    3.3.2) Examples:....15
4) Optional flags....16
  4.1) Alignment Algorithm....16
  4.2) Gap penalty schemes....16
  4.3) Output files....17
5) File types for similarity matrices, scales and profiles....18
  5.1) Similarity (substitution) matrices....18
  5.2) Position Specific Substitution Matrices....18
  5.3) Scales (e.g. hydrophobicity)....19
  5.4) Profiles....19
6) Description of sliding window types....20
7) Overview of all flags....22
8) References....24

1) Introduction

AlignMe is a program that allows the user to perform pair-wise alignments based on two sequences (section 3.1), two multiple sequence alignments (section 3.2) or two profiles (section 3.3) using a Needleman-Wunsch algorithm. Similarity ($Sim_{i,j}$) between two amino acids (I and J) may be measured by their evolutionary relationship based on a similarity matrix ($S_{A,B}$) or by differences of their hydrophobicity or predicted structural values ($|V_a - V_b|$). AlignMe is especially powerful because users can combine any number of (hydrophobicity) profiles (n) with any number of substitution matrices (m) with different weights (w) to guide the alignment (section 2.1.1)

$$Sim_{I,J} = \sum_{m}^{M}\left(w_m * S_{I,J}\right) - \sum_{n}^{N}\left(w_n * \left(\left|V_I - V_J\right|\right)\right)$$

This similarity measure is calculated for all amino acid pairs of two sequences in the Needleman-Wunsch algorithm. Additionally the user can choose between several gap penalty schemes for the treatment of mismatching stretches (section 2.1.d).

Alignment parameters for AlignMe have been optimized for alpha-helical membrane proteins with a combination of a position specific matrix (PSSM), a secondary structure structure prediction (PSIPRED) and a membrane prediction (OCTOPUS) resulting in highly accurate alignments. On our website http://www.bioinfo.mpg.de/AlignMe you can easily generate alignments using those optimized parameters! However, it is also possible to use AlignMe locally on your own computer, but therefore you need files containing secondary structure and membrane predictions as well as PSSMs on your local machine.

2) Using AlignMe with optimized parameters

Alignment parameters for AlignMe have been optimized for α-helical membrane proteins with a combination of a position specific substitution matrix (PSSM), a secondary structure prediction (PSIPRED) and a membrane prediction (OCTOPUS) resulting in highly accurate alignments. On our website http://www.bioinfo.mpg.de/AlignMe you can easily generate alignments using those optimized parameters! However, it is also possible to generate those alignments locally on your own computer but therefore you need locally files containing secondary structure predictions, files containing membrane predictions and PSSMs.

Position Specific Matrices can be obtained by a PSI-BLAST search with the following parameters:
[BLASTPGP] –b 0 – j 3 –h 0.001 –d [DATABASE] –i [INPUTFILE] –Q [PSSMFILE]
PSI-BLAST can be downloaded from ftp://ftp.ncbi.nlm.nih.gov/blast/executables/release/

Secondary structure predictions of PSIPRED can be generated locally if you install PSIPRED3.2 on your computer, which can be downloaded from:
http://bioinfadmin.cs.ucl.ac.uk/downloads/psipred/
For AlignMe, the .ss2 files of the PSIPRED prediction were used!

Membrane predictions of OCTOPUS can be obtained on their website:
http://octopus.cbr.su.se/
For AlignMe, you need the OCTOPUS network file (.nnprf text file)

Instructions on how to install all these 3 programs locally so that they can be easily used in combination with AlignMe are available upon request. Please write an email to AlignMe@rzg.mpg.de and we will help you to set up those programs and AlignMe locally on your computer. The databases that we have used in our study are available at http://bioinfo.mpg.de/AlignMe/download/ and are recommended to be used for this purpose!

If you have generated those predictions, you can use the perl script „use_best_parameters.pl" which is provided in the AlignMe download package. This script generates all necessary inputs (flags, similarity_score_file , gap penalties) that are required to generate optimized alignments with AlignMe using the 3 different inputs mentioned above. The following flags have to be provided for using the script "best_parameters.pl":
-alignme_exe <executable of AlignMe1.1>
-fasta1 <filename>
-fasta2 <filename>
-sspred1 <filename>
-sspred2 <filename>
-tmpred1 <filename>
-tmpred2 <filename>
-pssm1 <filename>
-pssm2 <filename>
-output_alignment <filename>
-output_profile <filename>

On our website, the plots of the aligned profiles were generated using gnuplot:
http://www.gnuplot.info/

3) Required flags

AlignMe is a very flexible program and thus, not only optimized parameters are provided – you also have the opportunity to generate custom alignments with your inputs (substitution matrices, predictions and so on) and gap penalties! Please keep in mind that the parameters that you provide to AlignMe have to fit to the proteins that you want to align.

Start the program by calling the `alignme1.1.exe` executable from a terminal. The program offers a variety of flags that are explained in this manual, and that can be provided in any order. They start with the usual '–' symbol and expect a filename or a value to follow. There are required flags that have no default values defined and that will cause error messages when missing. Optional flags have default values, and these are printed as warnings when the flag is not given. Look at section 4 to get more information about warnings. Type '–help' after the executable for basic usage information.

First of all, there are some required flags that have to be set by the user for AlignMe to run. For these flags no default values are defined. If one of the required flags is missing, you will receive a corresponding error message. Optional flags are described in section 4.

All modes of AlignMe require a so-called "similarity score file". This file contains information about the similarity measures that you wish to use to construct your alignment. These can be any combination of sequence, scale or profile similarity.

Each row in the similarity score file must start with a weight, which describes the influence of the chosen parameter on the alignment. If you are using only one type of similarity measure, then this value should be 1.0. An example of a similarity score file for an alignment based on a substitution matrix follows:

matrix.txt

```
weight: 1.0 type: SequenceSimilarity file: ./examples/matrices/blosum62.mat
```

More details about similarity score files are provided below.

3.1) Pair-wise sequence-to-sequence alignments

This section refers to alignments based on two protein sequences provided by the user. The sequence properties used for aligning the sequences can vary. For such an alignment two sequence files and a similarity score file must be provided.

The flags for the fasta files are:

–fasta_file1 <filename1>
–fasta_file2 <filename2>

The two amino acid sequences that you want to align must be in fasta format and in separate files (filename1 and filename2). For fasta format the first line of each file has to start with '>' symbol followed by a header (which is usually the name of the sequence but can also be left blank). All other text in these files is taken to be the amino acid sequence. If more than one '>' symbol is found, only the sequence following the first '>' symbol will be used, and an appropriate warning will be given.

Example of a file in fasta format:

```
1H2S_A.fa
>1H2S.pdb CHAIN_ID: A
MVGLTTLFWLGAIGMLVGTLAFAWAGRDAGSGERRYYVTLVGISGIAAVAYVVMALGVGWVPVAERTVFAPRYIDWILT
TPLIVYFLGLLAGLDSREFGIVITLNTVVMLAGFAGAMVPGIERYALFGMGAVAFLGLVYYLVGPMTESASQRSSGIKS
LYVRLRNLTVILWAIYPFIWLLGPPGVALLTPTVDVALIVYLDLVTKVGFGFIALDAAATLRAEHGE
```

–similarity_score_file <filename>

After this flag you have to provide filename of a file containing information about the type of alignment you wish to create. This file can be set up in 3 different ways depending on the type of alignment: (a) SequenceSimilarity, (b) ScaleSimilarity or (c) ProfileSimilarity or (d) any combination of (a), (b) and (c):

a) Similarity Score File for a pair-wise sequence alignment based on similarity matrices:

When using similarity matrices the corresponding line in the similarity_score_file should have the following format:
Weight <double> type SequenceSimilarity file <filename>

An example of a similarity score file for an alignment based on a matrix:

matrix.txt

```
weight: 1.0 type: SequenceSimilarity file: ./examples/matrices/blosum62.mat
```

The word following “type” describes the kind of alignment you wish to create. In this case SequenceSimilarity creates an alignment based on a substitution matrix. The filename has to contain the complete address of the file containing the matrix you want to use (only relative paths are used in the examples). An example substitution matrix is given in section 4.1.

b) Similarity Score File for a pair-wise sequence alignment based on Position Specific Matrices

Weight <double> type PostionSpecificSimilarity PSSM1 <filename> PSSM2 <filename>

An example of a similarity score file for an alignment based on Position Specific Matrices:

PSSM.txt

```
weight: 1.0 type: PositionSpecificSimilarity PSSM1: ./examples/PSSMs/1H2S.pssm PSSM2: ./examples/PSSMs/2EI4.pssm
```

The type PositionSpecificSimilarity is used to generated alignments based on the PSSMs which are provided subsequently. The file of PSSM1 has to be based on the sequence provided by the flag -fasta_file1 and the file after PSSM2 has to correspond to the sequence of the flag –fasta_file2.

The principle of PositionSpecificSimilarity for the replacement of amino acid A from sequence 1 by amino acid B from sequence 2 is: From PSSM1 the value for the replacement of A with B is taken, from PSSM2 the value for the replacement of B with A is taken and then the average of those values is calculated (their sum divided by 2).

Moreover, there is another method of using PSSMs for alignments supported by AlignMe called “PositionSpecificSimilarity”. The syntax is similar to those of “PositionSpecificSimilarity”:

simscore_PSSMprofile.txt

```
weight: 1.0 type: ProfilePositionSpecificSimilarity PSSM1: examples_best/1KPL.pssm PSSM2: examples_best/1OTS.pssm
```

However, the calculation is different. From PSSM1 all 20 values of amino acid A are compared to the corresponding values of amino acid B in PSSM1 (i.e., likeliness of A->A of PSSM1 with B-> A of PSSM2, A->C of 1, B->C of 2 and so on), their differences are summed up and then this value is divided by 20 (= number of amino acids types).

c) Similarity Score File for a pair-wise sequence alignment based on scales (e.g. hydrophobicity):

When using scales the corresponding line in the similarity_score_file should have the following format:

Weight <double> type ScaleSimilarity file <filename> windowtype <string> windowsize <integer>

An example of a similarity score file for an alignment based on a matrix:

scale.txt

```
weight: 1.0 type: ScaleSimilarity file: ./examples/scales/KD.txt windowtype: triangular windowsize: 13
```

The type ScaleSimilarity is used to create an alignment based on a scale. The filename refers to the file containing the scale you wish to use. In such a scale a value is assigned for all 20 amino acid types (see section 4.2 for format details). The similarity between two amino acids is calculated as the difference between their scale values.

Currently, 5 different window types are supported in AlignMe. These windows provide the option to smooth the scale values by averaging the values over a subsequence. These are: none, rectangular, triangular, sinoid, and zigzag (see section 5 for detailed information).

d) Similarity Score File for a pair-wise sequence alignment based on profiles (e.g. secondary structure predictions)

When aligning using profiles, the similarity_score_file should have the following format:
Weight <double> type UniversalProfileSimilarity column <double> headerlines <double> profile1 <filename1> profile2 <filename2>

An example of a similarity score file for an alignment based on profiles:

```
profile.txt
weight: 1.0 type: UniversalProfileSimilarity column: 5 headerlines: 1 profile1: ./examples/profiles/1H2S A.ss2 profile2: ./examples/profiles/2EI4 A.ss2
```

Here, the type UniversalProfileSimilarity is used in order to align user-specified profiles. A profile contains values in a certain column corresponding to a certain amino acid of the sequence (see section 4.3 for format). Therefore, the profiles must have the same length as the sequences. If the length of one of the profiles does not match that of the corresponding sequence (i.e., profile1 corresponds to fasta_file1, and profile2 to fasta_file2), an error message will be given. In addition, the column number from which the values will be taken must be provided. The number given after the tag “headerlines” describes the number of lines that will be skipped at the beginning of the profile-file. This option is useful if there are comments or other information at the beginning of the profile file that you do not want to include in the alignment.

e) Similarity Score File for a pair-wise sequence alignment based on a combination of matrices, scales or profiles

AlignMe allows combinations of input types. Each input type is defined in a separate row of the similarity_score_file. The following is an example of a similarity score file containing a combination of inputs:

```
combined.txt
weight: 1.0 type: SequenceSimilarity file: ./examples/matrices/blosum62.mat
weight: 1.0 type: ScaleSimilarity file: ./examples/scales/KD.txt windowtype: triangular windowsize: 13
weight: 1.0 type: UniversalProfileSimilarity column: 5 headerlines: 1 profile1: ./examples/profiles/1H2S_A.ss2 profile2: ./examples/profiles/2EI4_A.ss2
```

Basic Tutorial for Pair-wise Alignments

Here, we acquaint you with the basic commands of AlignMe with some examples using required flags. More information concerning optional flags is explained in section 3.

Change directory to the main AlignMe folder to run the following commands.

A) Pair-wise alignment of 2 sequences based on a substitution matrix

/alignme1.1.exe –fasta_file1 ./examples/fastas/1H2S_A.fa –fasta_file2 ./examples/fastas/2EI4_A.fa –similarity_score_file ./examples/similarity_scorefiles/matrix.txt

You will receive the following standard warning messages indicating the usage of default values:
No gap opening penalty provided. The default value 10 will be used.
No gap extension penalty provided. The default value 1 will be used.
No termini extension penalty provided. The default value 1 will be used.
No termini opening penalty provided. The default value will be used.
You did not provide a filename for the output of the sequence alignment. It will be written to aligned_sequences.aln

To modify the default values and to define custom output files, see section 4.

Only these 3 basic input flags that you have used to create this alignment are required flags. The alignment is now stored in aligned_sequences.aln. This alignment has been created based on the BLOSUM substitution matrix.

B) Pair-wise alignment of 2 sequences based on a PositionSpecificSubstitutionMatrix (PSSM)

/alignme1.1.exe –fasta_file1 ./examples/fastas/1H2S_A.fa –fasta_file2 ./examples/fastas/2EI4_A.fa –similarity_score_file ./examples/similarity_scorefiles/PSSM.txt –output_aligned_profiles my_aligned_PSSMs.aln

With this command, an alignment is generated based on "PositionSpecificSimilarity" and the aligned sequences are stored in the file "*my_aligned_PSSMs.aln*".

However, there is also the scoring type "ProfilePositionSpecificSimilarity" available, which has been described in section 3.1 and can be used with the following command:

/alignme1.1.exe –fasta_file1 ./examples/fastas/1H2S_A.fa –fasta_file2 ./examples/fastas/2EI4_A.fa –similarity_score_file ./examples/similarity_scorefiles/PSSMprofile.txt –output_aligned_profiles my_aligned_profile PSSMs.aln

Note that only the file after the flag "*–similarity_score_file"* is different!

C) Pair-wise alignment of 2 sequences based on a hydrophobicity scale

alignme1.1.exe –fasta_file1 ./examples/fastas/1H2S_A.fa –fasta_file2 ./examples/fastas/2EI4_A.fa –similarity_score_file ./examples/similarity_scorefiles/scale.txt

When creating alignments based on scales or profiles, it can be useful to use the optional flag –output_aligned_profiles (see section 4 for detailed information) to create an additional output file containing the aligned values of each sequence position. For example:

alignme1.1.exe –fasta_file1 ./examples/fastas/1H2S_A.fa –fasta_file2 ./examples/fastas/2EI4_A.fa –similarity_score_file ./examples/similarity_scorefiles/scale.txt –output_aligned_profiles my_aligned_profiles.aln

The aligned profiles are now written to my_aligned_profiles.aln, while the sequence alignment is still written to aligned_sequences.aln. To get a better overview of the underlying hydrophobicity of your sequence, the profile file can then be plotted, e.g. with xmgrace or gnuplot.

D) Pair-wise alignment of 2 sequences based on secondary-structure predictions

In this example two per-residue predictions are aligned with each other. These predictions were obtained with the secondary structure predictor PsiPred, but you can use any kind of program that creates a profile (i.e., transmembrane predictors, secondary structure predictors etc.).

alignme1.1.exe –fasta_file1 ./examples/fastas/1H2S_A.fa –fasta_file2 –similarity_score_file ./examples/similarity_scorfiles/profile.txt –output_aligned_profiles my_aligned_profiles.aln

E) Pair-wise alignment of 2 sequences based on combined inputs

alignme1.1.exe –fasta_file1 ./examples/1KPL.fa –fasta_file2 ./examples/1OTS.fa \ –similarity_score_file ./examples/similarity_scorefiles/combined.txt –output_aligned_profiles my_aligned_profiles.aln

This alignment is built using three different input types, i.e. amino-acid substitution (matrix), hydrophobicity (scale) and secondary structure (profile). For difficult alignments involving sequences with low sequence similarity, such combinations are usually more accurate than one input alone.

3.2) Alignment of two Multiple Sequence Alignments

This section describes alignments based on two multiple sequence alignments (MSAs) provided by the user. Currently, MSAs can only be used with scales. The scale values are averaged to generate an averaged profile, and then the two profiles are pair-wise aligned. This method is also referred to as family-averaged profile alignment.

The flags for the input alignments are:
–msa_file1 <filename1>
–msa_file2 <filename2>
The sequences you want to align must be in fasta format in separate files (filename1 and filename2). Each of these files must contain one or more sequences. Each sequence in the file must start with the ‘>’ symbol, followed by a header (it can also be left empty). All sequences of a file need to have the same length (including gaps), because it is assumed that these sequences are all aligned with each other. At least one sequence has to be provided per file.

–fraction_allowed_gaps <double>
Each column of the multiple sequence alignment is checked for the fraction of gaps that it contains. If the fraction of gaps in a given column is higher than this “fraction of gaps“ threshold value, this column will not be considered in the alignment. Default value = 0.5, i.e. columns in which more than 50 % of all positions are gaps are skipped and are not considered in the alignment.

–similarity_score_file <filename>
This flag requires you to provide a file containing information about the type of alignment you want to do. Currently, only alignments based on (hydrophobicity) scales, and using a triangular window are supported for alignment of two averaged multiple sequence alignments.

3.2.1) Similarity Score File for an alignment of two MSAs

For aligning two MSAs the line in the similarity_score_file should have the following format:
Weight <double> type ScaleSimilarity file <filename> windowtype msa_triangular windowsize <integer>

A valid example of a similarity score file:

simscore_msa.txt ×

```
weight 1.0 type ScaleSimilarity file examples/KD.txt wind_type triangular_msa size 13
```

In the above example the filename refers to the file containing the scale with which you wish to create an alignment. If amino acids of the submitted sequences are not in the corresponding scale, an error message will be given.

In the current version of AlignMe only the triangular_msa window type is supported (see section 5 for detailed information about sliding window types).

The length of the sequence must be longer than the chosen window size; otherwise the program will quit with an error.

3.2.2) Example

Enter the folder AlignMe main folder and test the following command:

*alignme1.1.exe –msa_file1 ./examples/bcct.fa –msa_file2 ./examples/deda.fa *
–similarity_score_file ./examples/simscore_msa.txt –fraction_allowed_gaps 0.5

Warnings are shown about default values; these can be ignored. Take a look at the aligned profiles, which have been written by default to aligned_profiles.aln.

3.3) Pair-wise profile-to-profile alignments

This section is about alignments based only on profiles. In contrast to approaches discussed in sections 2.1 and 2.2, you must not provide an amino acid sequence, which improves the speed of the alignment. Moreover, this option allows alignments of any kind of profile and is therefore not restricted to sequence alignments.

3.3.1) Similarity Score File for a pairwise profile-to-profile alignment

–similarity_score_file <filename>
For an alignment without sequences you only can use the type UniversalProfileSimilarity:

The similarity_score_file has to look like:
Weight <double> type UniversalProfileSimilarity column <double> headerlines <double> profile1 <filename1> profile2 <filename2>

Two profiles have to be provided. A profile contains corresponding values in a certain column (for more details see section 1) and you have to choose the column that will be used. Headerlines describes the number of lines that will be skipped at the beginning of the profile-file. This option is useful if there are comments or other information at the beginning of a file you do not want to include for the alignment.

3.3.2) Examples:

Enter the folder AlignMe main folder and test the following command:

alignme1.1.exe –similarity_score_file ./examples/similarity_score_files/profile.txt –output_aligned_profiles my_aligned_profiles.aln

You can have a look at your aligned profiles that will are stored in my_aligned_profiles.aln

4) Optional flags

4.1) Alignment Algorithm

–algorithm <name>

The user has option to change the algorithm being used for the alignment. Currently, only one option is possible, i.e. "global_affine", which is also the default value.

4.2) Gap penalty schemes

In any alignment two conditions exist, namely, sections of sequences match or they do not match. Mismatches are reflected by the introduction of gaps. Therefore, the quality of the alignment will be influenced not only by the similarity measures but also the criteria of introducing gaps; the latter are known as gap penalties. There are no "correct" values for gap penalties, instead they have to be adjusted by experience or optimization based on knowledge of "correct" alignments. Correspondingly, AlignMe has a variety of flags to control gap penalties.

The basic gap penalty scheme consists of two different penalties:

–gap_opening_penalty <value>

–gap_extension_penalty <value>

Typically, alignments with fewer and longer gaps are preferred over many short gaps. Therefore to open a new gap is usually assigned a higher penalty than to extend an existing gap. By default a gap opening penalty of 10 and a gap extension penalty of 1 are used. Different values can be set using these flags. Both values must be positive integers or fractions (no commas).

This basic scheme can be extended by the use of 2 additional gap penalties, which control gaps at the ends (N- and C-termini) of the sequences:

–termini_gap_opening_penalty <value>

–termini_gap_extension_penalty <value>

The latter flags allow gaps at the end of a sequence to be treated differently from gaps within the sequence. If the lengths of the sequences differ significantly it is probably useful to assign lower penalties to termini regions. If these values are not set, then by default they are assigned the same values as gap_open_penalty and gap_extension_penalty. Again, both values must be positive integers or fractions (no commas).

Finally, an advanced penalty scheme can be introduced using the flags:

–below_threshold_gap_opening_penalty <value>
–below_threshold_gap_extension_penalty <value>
–above_threshold_gap_opening_penalty <value>
–above_threshold_gap_extension_penalty <value>
–thresholds_for_penalties <value> <value> .....
With the last flag one can define thresholds for the scales or profiles. The other four flags then allow gaps to be distinguished if they are to be opened in regions where the profile values are above or below the thresholds. This scheme can be used to prevent gap insertion in secondary structure elements or transmembrane regions. For example: if a secondary structure prediction determines a helix probability above 0.5 one can avoid the introduction of gaps in that helix by defining a threshold of 0.5 and by providing larger values for the 'above' penalties than for the 'below' penalties.

Note that the values passed to the threshold flag have to match the definitions in the similarity score file. For example: '–thresholds_for_penalties 0.5 0.5' will apply these thresholds and the according gap penalties to the first two scales/profiles defined in the similarity score file, e.g. helix and hydrophobicity probabilities. Any scale/profile/matrix defined later in the similarity score file, (e.g. coil probability and hydrophobicity) will not be subject to this penalty scheme. In addition, if these five flags are provided, both termini gap penalties must also be provided.

4.3) Output files

–output_aligned_sequences <filename>
Provide the name of a file to which the alignment will be written. By default the alignment is written to a file called "aligned_sequences.aln" when generating a sequence-to-sequence alignment.

–output_aligned_profiles <filename>
Provide the name of a file to which an alignment of the input profiles will be written. By default the alignment will be written to a file called "aligned_profiles.aln" when aligning averaged multiple sequence alignments.

–profile_gap_value_for_plotting <value>
By default gaps will be written as a "?0" in the output profile alignment. This option is particularly useful for plotting your aligned sequences including gaps (e.g., with gnuplot or the attached perl-script "reformat_profile_output.pl" and xmgrace).

5) File types for similarity matrices, scales and profiles

5.1) Similarity (substitution) matrices

To align sequences in the traditional manner, i.e. with the SequenceSimilarity option, requires a matrix that defines the likelihood of substituting one amino acid type with another. The following is an example of a matrix file in the correct format (i.e. BLOSUM62):

blosum62.txt

```
   A  C  D  E  F  G  H  I  K  L  M  N  P  Q  R  S  T  V  W  Y
A  4  0 -2 -1 -2  0 -2 -1 -1 -1 -1 -2 -1 -1 -1  1  0  0 -3 -2
C  0  9 -3 -4 -2 -3 -3 -1 -3 -1 -1 -3 -3 -3 -3 -1 -1 -1 -2 -2
D -2 -3  6  2 -3 -1 -1 -3 -1 -4 -3  1 -1  0 -2  0 -1 -3 -4 -3
E -1 -4  2  5 -3 -2  0 -3  1 -3 -2  0 -1  2  0  0 -1 -2 -3 -2
F -2 -2 -3 -3  6 -3 -1  0 -3  0  0 -3 -4 -3 -3 -2 -2 -1  1  3
G  0 -3 -1 -2 -3  6 -2 -4 -2 -4 -3  0 -2 -2 -2  0 -2 -3 -2 -3
H -2 -3 -1  0 -1 -2  8 -3 -1 -3 -2  1 -2  0  0 -1 -2 -3 -2  2
I -1 -1 -3 -3  0 -4 -3  4 -3  2  1 -3 -3 -3 -3 -2 -1  3 -3 -1
K -1 -3 -1  1 -3 -2 -1 -3  5 -2 -1  0 -1  1  2  0 -1 -2 -3 -2
L -1 -1 -4 -3  0 -4 -3  2 -2  4  2 -3 -3 -2 -2 -2 -1  1 -2 -1
M -1 -1 -3 -2  0 -3 -2  1 -1  2  5 -2 -2  0 -1 -1 -1  1 -1 -1
N -2 -3  1  0 -3  0  1 -3  0 -3 -2  6 -2  0  0  1  0 -3 -4 -2
P -1 -3 -1 -1 -4 -2 -2 -3 -1 -3 -2 -2  7 -1 -2 -1 -1 -2 -4 -3
Q -1 -3  0  2 -3 -2  0 -3  1 -2  0  0 -1  5  1  0 -1 -2 -2 -1
R -1 -3 -2  0 -3 -2  0 -3  2 -2 -1  0 -2  1  5 -1 -1 -3 -3 -2
S  1 -1  0  0 -2  0 -1 -2  0 -2 -1  1 -1  0 -1  4  1 -2 -3 -2
T  0 -1 -1 -1 -2 -2 -2 -1 -1 -1 -1  0 -1 -1 -1  1  5  0 -2 -2
V  0 -1 -3 -2 -1 -3 -3  3 -2  1  1 -3 -2 -2 -3 -2  0  4 -3 -1
W -3 -2 -4 -3  1 -2 -2 -3 -3 -2 -1 -4 -4 -2 -3 -3 -2 -3 11  2
Y -2 -2 -3 -2  3 -3  2 -1 -2 -1 -1 -2 -3 -1 -2 -2 -2 -1  2  7
```

5.2) Position Specific Substitution Matrices

In contrast to general substitution matrices, Position Specific Substitution matrices have different substitution rates for each sequence position. The first column contains the number of the sequence position, the second column the amino acid at this positions and the following 20 columns are the rates of the amino acid from the second column to be replaced by one of the 20 standard amino acids (shown in line 2). Please note that AlignMe only supports PSSM that have a comment like „Last position-specific...“ in the first line, followed by the 20 standard amino acids in the second line and all other amino acids in the next lines:

1H2S_A.pssm

```
Last position-specific scoring matrix computed, weighted observed perc
           A  R  N  D  C  Q  E  G  H  I  L  K  M  F  P  S  T  W  Y  V
    1 M   -3 -3 -4 -5 -3 -2 -4 -5 -3  0  2 -3  9 -1 -4 -3 -2 -3 -3 -1
    2 V    0 -4  0  5 -4 -3 -2 -4 -4  2 -1 -3  2 -4 -4 -3 -3 -5 -4  2
    3 G    3 -4 -3  0 -4 -1 -1  2 -4 -3  1 -3 -3 -4  4  0  1 -5 -4 -3
    4 L   -3 -4 -3  1 -4 -3  2 -2  0  2  1 -3  1  0 -4 -1  2 -5 -3  3
    5 T    1 -3 -1 -3 -4  1 -2  2 -3 -3 -4 -3 -4  0 -4  5  2 -5  0 -4
    6 T   -2 -4  3  4 -4 -3 -3 -4 -4  3 -2 -3 -2 -2 -4  1  3 -5 -2  0
    7 L    1 -4 -4 -5 -4 -4 -4 -4 -4 -1  0 -4  0  0 -5  2  2  9  0  0
    8 F   -3 -5 -5 -6 -4 -1 -5 -5  0 -1  4 -5 -1  5 -6 -5 -4  1  4 -3
    9 W   -3 -3 -5 -6 -5 -4 -5 -1 -5 -3 -2 -5  0  2 -6  0 -1 11  1 -2
   10 L    2 -5 -5 -6 -4 -5 -5 -2 -5  3  3 -5 -2  2 -5 -3 -1 -5 -3  4
```

5.3) Scales (e.g. hydrophobicity)

For aligning two sequences according to a scale such as a hydrophobicity scale, the ScaleSimilarity mode is used. This requires an input file in which each of the two amino-acid types is assigned a value on the scale. Thus, the similarity between two amino acids is the difference between their scale values. The following is an example input scale file (i.e. Kyte-Doolittle hydrophobicity scale

KD.txt

```
A        1.80
C        2.50
D       -3.50
E       -3.50
F        2.80
G       -0.40
H       -3.20
I        4.50
K       -3.90
L        3.80
M        1.90
N       -3.50
P       -1.60
Q       -3.50
R       -4.50
S       -0.80
T       -0.70
V        4.20
W       -0.90
Y       -1.30
```

5.4) Profiles

AlignMe allows for alignments of any kind of profiles like secondary structure predictions obtained from PsiPred (left) or membrane predictions from OCTOPUS (right). The user has to choose the column from which the values should be taken from and it is possible to skip commented lines using the "headerlines" option in the *similarity_score_file*.

1H2S_A.ss2

```
# PSIPRED VFORMAT (PSIPRED V3.2)

   1 M C   1.000  0.000  0.000
   2 V C   0.548  0.318  0.015
   3 G H   0.287  0.783  0.034
   4 L H   0.144  0.902  0.039
   5 T H   0.088  0.947  0.011
   6 T H   0.038  0.975  0.003
   7 L H   0.002  0.997  0.002
   8 F H   0.006  0.994  0.001
   9 W H   0.003  0.995  0.001
  10 L H   0.005  0.993  0.001
```

1H2S_A.nnprf

```
Pos     M       L       G       R       i       o       AA
1       0.100   0.627   0.145   0.345   0.170   0.820   M
2       0.100   0.645   0.118   0.436   0.160   0.830   V
3       0.127   0.609   0.109   0.518   0.140   0.850   G
4       0.482   0.600   0.100   0.509   0.100   0.890   L
5       0.736   0.527   0.091   0.382   0.080   0.920   T
6       0.827   0.264   0.091   0.418   0.060   0.940   T
7       0.955   0.136   0.091   0.227   0.050   0.950   L
8       0.991   0.136   0.091   0.191   0.050   0.950   F
9       0.991   0.136   0.091   0.191   0.080   0.920   W
10      0.991   0.100   0.091   0.136   0.190   0.810   L
```

6) Description of sliding window types

Alignments with hydrophobicity scales allow the usage of sliding windows to generate a smoothed hydrophobicity profile that is not based on single amino acid positions only but also takes neighboring amino acids of a sequence-position into account. None, triangular, rectangular, sinusoidal and zigzag are also supported sliding window types for pairwise sequence-to-sequence alignments.

rectangular

In a rectangular window the (hydrophobicity) values of all amino acids within the window contribute equally to the total, which is then divided by the width of the window. This value is then assigned to the position at the window centre, so that the values of the neighboring residues are effectively used to smooth out the profile. This option may be used in combination with a 6 gap-penalty set to subdivide the sequence into two regions with different gap penalties according to a threshold value (see section 3.2).

triangular

The principle of a triangular window is similar to that of a rectangular window but emphasizes the value associated with the amino acid at the centre of the window, whereas the contribution of values either side decreases linearly from the central position. Thus, profiles become smoothed, but less drastically than in the case of the rectangular window. This option is may be useful in combination with a 6 gap-penalty set and a threshold value (see section 3.2).

sinusoidal

In sinusoidal averaging, the contribution of amino-acid positions either side of the central position will vary according to a sine wave. Thus, every 3.4th amino acid will contribute fully to the averaging, whereas every 1.7th amino acid contributes nothing to the averaging. Such sinusoidal window-averaging may be useful for aligning proteins consisting mainly of alpha helices because it mimics the periodicity of amino acid properties in a helix. Thus, for a position that is on a hydrophobic face of a helix, its score will be averaged along with other hydrophobic residues, but not with the hydrophilic residues in-between.

zigzag

A zigzag window tries to imitate the alternating hydrophobicity pattern of a beta-sheet (i.e. one side is hydrophobic and the other hydrophilic). Starting from the window-centre (i) every second amino acid contributes to the averaging of the central residue.

Example for a window size of 5:

Positions i-2, i, i-2 contribute to the average, whereas positions i-1 and i+1 have no effect on the average.

Currently, only one averaging type is supported for alignments of averaged multiple sequence alignments:

triangular_msa

To create average hydropathy profiles based on the MSAs, a triangular sliding window can be used. There are two main steps in this procedure.

1) For every sequence in the MSA its own window-averaged hydropathy profile is calculated first. Due to a presence of gaps in the MSA, the sliding window in this case is flexible, i.e. its length can be extended by a number of gaps found on every side of the window, so that the window of a specified length N still covers N non-gappy positions.

2) After average hydropathy profiles have been calculated for every sequence in the MSA, the average hydropathy profile of the entire MSA is calculated by averaging hydrophobicity values at every position in the MSA. At every position of the MSA hydrophobicity values are summed up, while gaps are ignored. Then the total sum at every position of the MSA is divided by the number of non-gaps.

7) Overview of all flags

Flags for input files:

–similarity_score_file [file]	file containing information about the type of alignment you want to do
–fasta_file1 [file]	file containing an amino acid sequence in fasta format
–fasta_file2 [file]	file containing an amino acid sequence in fasta format
–msa_file1 [file]	file containing a multiple sequence alignment in which all sequences are the same length
–msa_file2 [file]	file containing a multiple sequence alignment in which all sequences are the same length

–similarity_score_file must always be provided
–fasta_file1 and –fasta_file2 must be provided together
The same is true for –msa_file1 and msa_file2.

Flags for output files:

–output_aligned_sequences [file]	file to which the aligned amino acid sequences are printed
–output_aligned_profiles [file]	file to which the aligned profile values are printed
-extract_from_MSA_sequences_with_ids [value1] [value2]	from each averaged multiple sequence alignment a sequence is extraced. Value1 is i-th sequence from msa_file1 and value2 the j-th sequence from msa_file2
-output_extracted_sequences [file]	file to which the sequences that are extracted from the averaged MSA are written to

You can choose one of the following gap penalty sets.
A combination is not possible!

Flags for a set of 2 gap penalties:

–gap_opening_penalty [value]	penalty for opening gaps
–gap_extension_penalty [value]	penalty for extending gaps

Flags for a set of 4 gap penalties:

–gap_opening_penalty [value]	penalty for opening gaps within the sequence
–gap_extension_penalty [value]	penalty for extending gaps within the sequence
–termini_gap_opening_penalty [value]	additional penalty for opening gaps at the end of the sequence
–termini_gap_extension_penalty [value]	additional penalty for extending gaps at the end of the sequence

Flags for a set of 6 gap penalties:

–thresholds_for_penalties [values]	defines a threshold according to which gap penalties are assigned to a given position of the sequence
–below_threshold_gap_opening_penalty [value]	penalty for opening gaps opposite to residues with a value below the chosen threshold
–below_threshold_gap_extension_penalty [value]	penalty for extending gaps opposite to residues with a value below the chosen threshold
–above_threshold_gap_opening_penalty [value]	penalty for opening gaps opposite to residues with a value above the chosen threshold
–above_threshold_gap_extension_penalty [value]	penalty for extending gaps opposite to residues with a value above the chosen threshold
–termini_gap_opening_penalty [value]	additional penalty for opening gaps at the end of the sequence
–termini_gap_extension_penalty [value]	additional penalty for extending gaps at the end of the sequence

Other flags

–fraction_allowed_gaps [value]	columns of the MSA in which more than [value] positions are gaps are skipped and not considered in the alignment
–profile_gap_value_for_plotting [value]	define a value to be assigned to gaps in the output aligned profiles, the standard value for gaps is ?0
- alignment_output_format	Formatting type of the alignment to be generated. Allowed types are: ClustalW and fasta. If this flag is not provided, alignments are written in ClustalW format.

8) References

If you have used AlignMe for your research and study, please cite this reference:

Khafizov, F., Staritzbichler, R., Stamm, M. and L.R. Forrest. A study of the evolution of inverted-topology repeats from LeuT-fold transporters using AlignMe. *Biochemistry*, (2010)

A webserver of AlignMe is available at:
http://www.forrestlab.org/AlignMe/
